# The relationship between traditional Chinese medicine constitution and indexes in chronic obstructive pulmonary disease patients: a systematic review and network meta-analysis

**DOI:** 10.3389/fmed.2026.1815235

**Published:** 2026-05-22

**Authors:** Sheng Xie, Meiling Xie, Guanhong Li, Yu Zhang, Hui Wang, Yuqiong Zheng

**Affiliations:** 1 Department of Pulmonary and Critical Care Medicine, Chengdu First People’s Hospital, Sichuan, China; 2 Department of Traditional Chinese Medicine, Sichuan Electric Power Hospital, Sichuan, China

**Keywords:** COPD, lung function, network meta-analysis, phenotype, TCM constitution

## Abstract

**Objective:**

To investigate the characteristics of Chronic Obstructive Pulmonary Disease (COPD) patients with different Traditional Chinese Medicine (TCM) constitutions by analyzing the differences of indexes (smoking index, ratio of forced expiratory volume in 1 sec and forced vital capacity (FEV1/FVC),forced expiratory volume in 1 sec % predicted (FEV1%Pred), number of acute exacerbations per year (AE/y) and COPD assessment test (CAT)) in COPD patients with different TCM constitutions.

**Methods:**

Literatures were retrieved from PubMed, Embase (Ovid platform), Cochrane Library, CNKI, VIP, Wanfang Database, and Sinomed, eligible literature was screened. Quality assessment was performed using the Agency for Healthcare Research and Quality (AHRQ) checklist and the Newcastle-Ottawa Scale (NOS). Network meta-analysis was conducted using Stata and Addis software for data analysis. The study was registered with PROSPERO, https://www.crd.york.ac.uk/PROSPERO/view/CRD42023433078.

**Results:**

A total of 11 studies, which contain 13 references, with 2,168 participants were included. Network Meta-analysis revealed that in COPD patients, the top two TCM constitutions with higher smoking index were yang-deficiency and qi-deficiency; the top two TCM constitutions with low FEV1/FVC were yang-deficiency and qi-deficiency; the top three TCM constitutions with low FEV1%Pred were yang-deficiency, blood-stasis and qi-deficiency; the top three TCM constitutions with higher acute exacerbate numbers per year and CAT scores were yang-deficiency, blood-stasis and qi-deficiency.

**Conclusion:**

The indexes of COPD patients with different TCM constitutions are varying. The indexes of yang-deficiency and qi-deficiency are always among the worst group, and other indexes differed from each other. The result can help clinicians in prevention, evaluation, treatment and rehabilitation decision-making.

**Systematic review registration:**

https://www.crd.york.ac.uk/PROSPERO/view/CRD42023433078, identifier (CRD42023433078).

## 1 Introduction

Chronic Obstructive Pulmonary Disease (COPD) is a global chronic respiratory disease with a long history. Its global prevalence was 10.3% (95% confidence interval (CI): 8.2, 12.8%), resulting in more than 3 million deaths worldwide (2012) and more than 0.91 million deaths in China (2013), and this number has been increasing over the years (1–4). COPD is also associated with significant social and economic burdens globally (1, 41). For better evaluation, treatment and prognosis assessment of COPD, there are several classifications methods existed, including the GOLD Grades based on spirometer results, GOLD ABE assessment based on exacerbation history and symptoms, and 4 main phenotypes based on clinical subgroups (1, 5). However, it should be noted that these classifications do possess certain limitations. For example, the forced spirometry should be done every time GOLD Grades are assessed.

Traditional Chinese Medicine (TCM) constitution refers to an individual’s comprehensive and relatively stable inherent characteristics of morphological structure, physiological functions and psychological state that are formed based on congenital inheritance and acquired feature during the process of human life (6). It’s a comprehensive summary of physical and mental characteristics, disease tendency and environmental adaptability within a population. TCM constitution can be assessed in both healthy people and patients, easy to access, and can be assessed right on the time of disease diagnosed and the follow visits, making its own advantages. However, decision-making based on TCM constitution has relied on personal experience over an extended period. Although several studies have investigated TCM constitution recently, including the studies on the relationship between TCM constitution and COPD, they were somehow limited in quantity and have their own shortcomings, such as small sample size and partially inconsistent results from each other. The aim of this study is to reveal the relationship between TCM constitution and indexes in COPD patients, and to assist clinicians in making a better decision on disease prevention, evaluation, treatment and rehabilitation.

## 2 Method

### 2.1 Study design

This systematic review and network meta-analysis was performed as part of the construction project of national famous TCM experts inheritance studio [Education Letter of Traditional Chinese Medicine (2022)75]. We registered our study on PROSPERO (CRD 42023433078) and followed the Preferred Reporting Items for Systematic Reviews and Meta-Analyses extension statement for network meta-analysis (PRISMA-NMA) (7).

### 2.2 Search strategy

We searched online academic platforms from inception to March 31th, 2026. The platforms included Cochrane Library, Embase (using Ovid platform), PubMed in English and CNKI, Wangfang database, VIP and SinoMed in Chinese. The search keywords included chronic obstructive pulmonary disease, COPD, body constitution of TCM, body constitution of traditional Chinese medicine, TCM body constitution, traditional Chinese medicine body constitution, constitution of TCM, constitution of traditional Chinese medicine, TCM constitution, traditional Chinese medicine constitution, constitution of CM, constitution of Chinese medicine, Chinese medicine constitution and CM constitution in English platforms, and 慢性阻塞性肺疾病、慢阻肺, 慢性阻塞性肺病, 慢性阻塞性肺部疾病 and 中医体质 in Chinese platforms. The fully process of retrieval included comprehensive literature retrieval, duplicated literature deleting, reading titles and abstracts to rough screening, carefully reading the rest full texts to deep screening, disputed literatures discussion, and eligible literature inclusion. The complete search strategy is presented in the Appendix.

### 2.3 Index selection

We selected five indexes in this study: smoking index, ratio of forced expiratory volume in 1 sec and forced vital capacity (FEV1/FVC), forced expiratory volume in 1 sec % predicted (FEV1%Pred), number of acute exacerbations per year (AE/y) and COPD assessment test (CAT). The smoking index is calculated by multiplying the number of cigarettes smoked per day by the number of years smoked. We chose this index because cigarette smoking is a key environmental risk factor for COPD (1). FVC is the volume of air forcibly exhaled from the point of maximal inspiration, FEV1 is the volume of air exhaled during the first second of this maneuver, and FEV1/FVC is the ratio of FEV1 and FVC. FEV1%Pred is calculated by comparing actual FEV1 value to reference value based on age, height and sex. We chose these two indexes for they are important spirometry values in COPD. FEV1/FVC detects the existence of airflow obstruction, and FEV1%Pred assesses its severity. AE/y is the average annual frequency of acute exacerbations, CAT is a questionnaire that assesses health status in COPD patients. We included the two indexes as they reflect disease severity. While AE/y is objective, CAT rely on patient-reported experiences.

### 2.4 Selection criteria

Inclusion criteria were listed as follows: 1. Participants should meet the diagnosis criteria of COPD; 2. The TCM constitution assessment should follow the items of TCM Constitution Classification and Judgment (ZYYXH/T157-2009); 3. Participants should be aged 18 years or older; 4. Participants should be assessed and their TCM constitutions should be determined, and at least one outcome would be grouped by this assessment; 5. One or more following outcomes should be included: smoking index, FEV1/FVC, FEV1%Pred, AE/y and CAT score, and these data can be extracted directly or after transforming; 6. Outcomes should be compared across the different TCM constitutions; 7. Observational studies (no intervention) were included; 8. The public language of literature should be Chinese or English.

Exclusion criteria were as follows: 1. Participants included pregnant or lactating women; 2. Data cannot be extracted (transformed or not); 3. The outcomes only contained one TCM constitution, no other TCM constitutions as comparisons; 4. Animal research.

### 2.5 Data extraction

Based on the aforementioned inclusion and exclusion criteria, two researchers (Sheng Xie and Meiling Xie) conducted literature retrieval, data extraction, and quality assessment independently. If any disputes arise during this process, they should be resolved by discussion. A third party (Guanhong Li, Yu Zhang, and Hui Wang) will made the final decision if no agreement was reached by discussion. Data of the study design, number of subjects, demographic baseline, duration and location of the study, primary TCM constitutions of the study, disease stage and outcomes were extracted and analyzed.

### 2.6 Quality assessment

Cross-sectional/prevalence study quality of the Agency for Healthcare Research and Quality (AHRQ) was adapted for the assessment of cross-sectional literature quality and risk of bias. It contains 11 items: 1. Define the source of information (survey, record review), 2. List inclusion and exclusion criteria for exposed and unexposed subjects (cases and controls) or refer to previous publications, 3. Indicate time period used for identifying patients, 4. Indicate whether or not subjects were consecutive if not population-based, 5. Indicate if evaluators of subjective components of study were masked to other aspects of the status of the participants, 6. Describe any assessments undertaken for quality assurance purposes (e.g., test/retest of primary outcome measurements), 7. Explain any patient exclusions from analysis, 8. Describe how confounding was assessed and/or controlled, 9. If applicable, explain how missing data were handled in the analysis, 10. Summarize patient response rates and completeness of data collection, 11. Clarify what follow-up, if any, was expected and the percentage of patients for which incomplete data or follow-up was obtained (8). According to the specific situation, the literature was evaluated as yes, no or uncertain in each item.

Newcastle - Ottawa quality assessment Scale (NOS) was adapted for the assessment of case control and cohort literature quality and risk of bias. It contains 3 items (selection, comparability and outcome) and 8 subitems (4 selections, 1 comparability and 3 outcomes). For case control studies, the subitems are: 1. Is the case definition adequate?, 2. Representativeness of the cases, 3. Selection of controls, 4. Definition of Controls, 5. Comparability of cases and controls on the basis of the design or analysis, 6. Ascertainment of exposure, 7. Same method of ascertainment for cases and controls, 8. Non-Response rate. For cohort studies, the subitems are: 1. Representativeness of the exposed cohort, 2. Selection of the non exposed cohort, 3. Ascertainment of exposure, 4. Demonstration that outcome of interest was not present at start of study, 5. Comparability of cohorts on the basis of the design or analysis, 6. Assessment of outcome, 7. Was follow-up long enough for outcomes to occur, 8. Adequacy of follow up of cohorts. A study can be awarded a maximum of one star for each numbered item within the Selection and Outcome categories. A maximum of two stars can be given for Comparability.

### 2.7 Data analysis

Given that upto 9 TCM constitutions were compared, conventional pair-wise meta-analysis would have introduced additional bias. Therefore, network meta-analysis (NMA) was chosen for statistical synthesis. However, as NMA is typically applied to interventional studies, the statistical approach adopted in this study is exploratory in nature, and the conclusions should be interpreted with strict caution. Software Addis 1.16.5 and Stata 13.0 were used for data analysis. All the outcomes of this study were continuous data and were represented by Mean Difference (MD) and 95% Confidence Interval (CI). Descriptive analysis was introduced when statistical analysis cannot be performed. There was no statistic difference of the compared TCM constitutions if the 95% CI of MD crossed 0. Direct meta-analysis was performed using Stata. Addis was used for network meta-analysis, using Bayesian model. Convergence of the model was assessed by Potential Scale Reduction Factor (PSRF). A PSRF close to 1 indicates approximate convergence has been reached. Since all the studies included were multi-arm studies, and the number of arms was 3 or more, the inconsistency test was not necessary and network consistency model was adopted. The ranking was established by both the results of the Surface under the Cumulative Ranking curve (SUCRA) and the league table. Comparison-adjusted funnel plot analysis was performed for publication bias. Sensitivity analyses, subgroup analyses, and network meta-regression will be performed as appropriate.

## 3 Result

### 3.1 Literature screening

**Figure 1:**
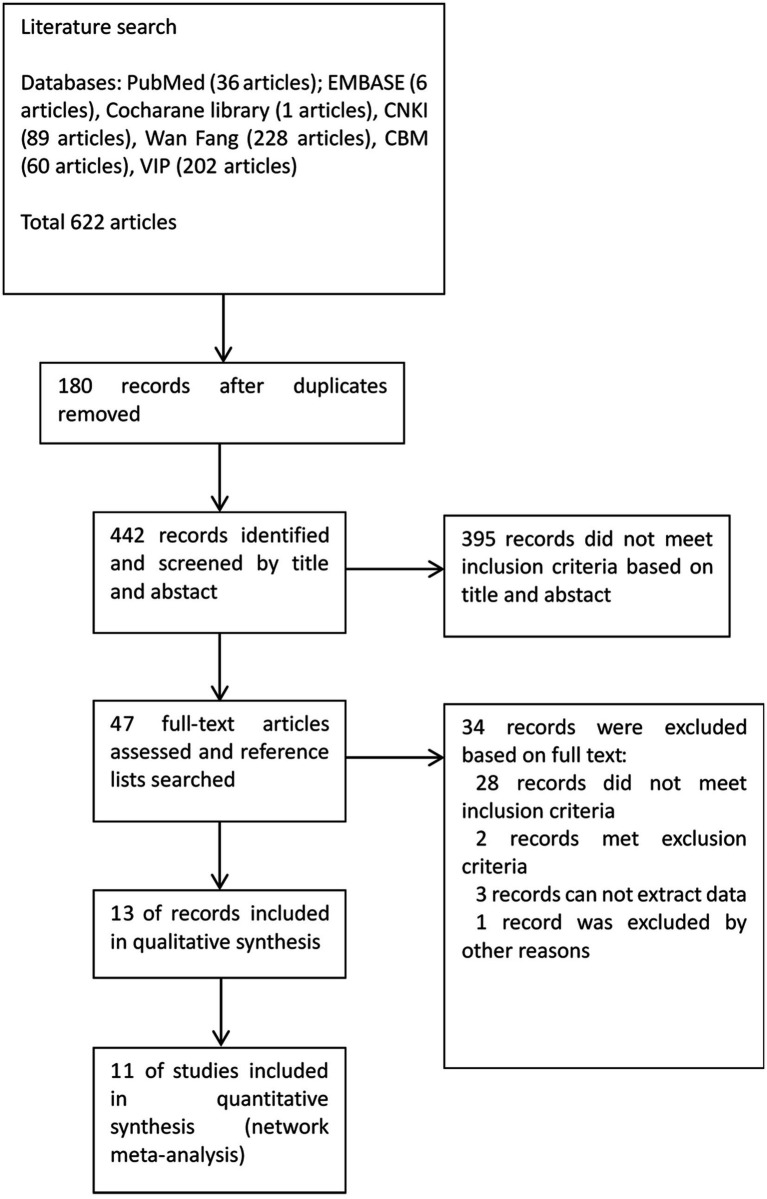
Literature screen process and outcomes.

After comprehensive retrieval of the databases, a total of 622 citations were identified, and 13 full manuscripts with 11 studies were included after screening (9–21). The process of retrieval screening is shown in Figure 1.

### 3.2 Characteristics and primary results of included citations

Of the 11 studies included, one was retrospective study (15), two were prospective observational study (16, 21), the remaining were cross-sectional studies. The articles were published between 2014 and 2023. A total of 2,168 adult participants were included. In all studies, the maximum number of subjects was 416 and the minimum was 44. The duration of these studies ranged from 8 to 52 months. Three studies did not specify the disease stage investigated (9, 19, 20), two reported the participants were in acute exacerbation stage (17, 18, 21), participants of other studies were in stable stage. The basic characteristics of the 10 studies are shown in Table 1, and the primary results are shown in Table 2.

**Table 1:** Characteristics of included studies.

Citation	Study design	Number of subjects	Age		Gender		Duration of study	Location of study	Disease stage	Primary TCM constitutions	Outcome indicators
					Male	Female					
Zhong et al. (9)	Cross-sectional	298	64.52 ± 13.63		163	135	2017.12–2021.3	Maoming hospital of Traditional Chinese Medicine	Unclear	Qi-deficiency, Yang-deficiency, Phlegm-dampness, specific-diathesis	Distribution, smoking index, Lung fuction, AE/y
Huang (10)	Cross-sectional	137	71.72 ± 7.42		123	14	2020.2–2021.2	Fuzhou Traditional Chinese Medicine hospital	Stable	Qi-deficiency, Yang-deficiency, Phlegm-dampness, Dampness-heat, Blood-stasis	Distribution, CAT score, age, course of disease, AE/y, BMI, smoking index, complication, influential factor
Wu (11)#	Cross-sectional	44	65.59 ± 11.85	71.58 ± 7.21	30	14	2019.11–2021.1	Respiratory clinic of Dongzhimen hospital Beijing university of Chinese Medicine	Stable	Gentleness, Qi-deficiency, Yang-deficiency, Phlegm-dampness	Age, Gender, BMI, smoking index, course of disease, AE/y, lung function, blood routine test, CAT score, mMRC score, TCM syptom scores for gastrointestinal disease, T-lymphocyte related indicators, correlation between T lymphocyte-related indicators and gastrointestinal score
Lu et al. (12)*#	Cross-sectional	81	65.62 ± 10.23	69.83 ± 7.63	64	17	2019.4–2019.12	Outpatient clinic of Suzhou hospital of Traditional Chinese Medicine and Suzhou high-tech zone Tongan health center	Stable	Gentleness, Qi-deficiency, Yang-deficiency, Phlegm-dampness	Lung fuction, CAT score, mMRC score, AE/y, peripheral T lymphocyte subset, immunoglobulin, related inflammatory factors, leptin, thyroid hormones
Lin (14)	Cross-sectional	190	65.67 ± 8.64		177	13	2016.11–2018.10	Inpatient and outpatient of respiratory department of Fujian provencial people’s hospital	Stable	Qi-deficiency, Yang-deficiency, Phlegm-dampness, Dampness-heat, Blood-stasis	Distribution, BODE index, GOLD grades, AE/y, smoking index, TG, TC, FIB, D-dimer, albumin
Gao et al. (15)	Retrospective	201	70.00 ± 5.27		103	98	2014.1–2016.12	Inpatient and outpatient of respiratory department of Wuxi Traditional Chinese Medicine hospital	Stable	Gentleness, Qi-deficiency, Yang-deficiency, Yin-deficiency, Phlegm-dampness, Blood-stasis, Qi-depression, Specific-diathesis	Distribution, AE/y
Liang et al. (16)	Prospective observational	169	55–75		128	41	2013.8–2015.8	Inpatient and outpatient of respiratory department of Guangdong Hospital of Chinese and Western Medicine and Shatou branch hospital	Stable	Qi-deficiency, Yin-deficiency, Phlegm-dampness, Dampness-heat	Distribution, mMRC score, CAT score
Wang et al. (17)*	Cross-sectional	218	68.77 ± 10.92		129	89	2012.1–2012.12	Inpatient of respiratory department of Shanxi provencial people’s hospital	Acute Exacerbation	Qi-deficiency, Yang-deficiency, Yin-deficiency, Blood-stasis	Distribution, age, gender, BMI, mMRC score, CAT score, lung fuction, CRP, blood gas analysis, cough up phlegm, course of disease
Lu et al. (19)	Cross-sectional	210	68.50 ± 8.04		156	54	2011.5–2012.4	Shuguang hospital and Gongli hospital of Shanghai university of traditional Chinese Medicine	Unclear	Qi-deficiency, Yang-deficiency, Yin-deficiency, Phlegm-dampness, Dampness-heat, Blood-stasis, Qi-depression, Specific-diathesis	Distribution, GOLD grade, BMI, gender, age
Wang et al. (20)	Cross-sectional	416	64.6 ± 12.5		294	122	2015.2–2019.6	Respiratory and critical care medicine department of Guangdong provencial hospital of Chinese medicine	Unclear	Qi-deficiency, Yang-deficiency, Yin-deficiency, Phlegm-dampness, Dampness-heat, Blood-stasis, Qi-depression	Distribution, TCM syndrome type and distribution, GOLD grade, BMI, age
Hou (21)	Prospective observational	204	69.25 ± 9.28		135	69	2021.11–2022.11	Department of pulmonary disease of Qinghai provincial hospital of traditional Chinese medicine	Acute exacerbation	Qi-deficiency, Yang-deficiency, Yin-deficiency, Phlegm-dampness, Dampness-heat, Blood-stasis	Distribution, Gender, Season of onset, age, duration of illness, BMI, smoking index, CRP, mMRC score

* The data were obtained from journal article and dissertation of the same study; # The ages in this study are shown as the youngest and oldest groups.

**Table 2:** Primary results of included studies, presented in descending order based on mean value.

Citation	Smoking index	FEV1/FVC	FEV1%Pred	AE/y	CAT
Zhong et al. (9)	Yang-d (693.12 ± 301.45), Qi-d (625.36 ± 308.26), Phl-d (346.74 ± 193.21), Spe-d (283.56 ± 122.31), *p* < 0.001	Phl-d (62.37 ± 14.62), Spe-d (59.02 ± 10.21), Qi-d (51.36 ± 12.56), Yang-d (47.53 ± 9.25), *p* < 0.001	Phl-d (69.39 ± 7.52), Spe-d (65.84 ± 5.93), Qi-d (56.36 ± 6.62), Yang-d (51.68 ± 5.93), *p* < 0.001	Yang-d (3.89 ± 1.52), Qi-d (3.10 ± 1.03), Phl-d (0.53 ± 0.12), Spe-d (0.31 ± 0.21), *p* < 0.001	–
Huang (10)	Yang-d (509.52 ± 367.46), Qi-d (446.15 ± 338.61), Dam-h (333.33 ± 246.18), Blo-s (305.26 ± 277.84), Phl-d (173.33 ± 237.45)*	–	–	Yang-d (2.61 ± 1.22), Blo-s (2.53 ± 1.39), Qi-d (2.02 ± 1.88), Dam-h (1.00 ± 1.65), Phl-d (0.93 ± 0.70), *p* < 0.05	Blo-s (24.58 ± 4.75), Yang-d (20.55 ± 5.62), Qi-d (18.38 ± 4.28), Phl-d (15.00 ± 4.63), Dam-h (14.5 ± 5.35)*
Wu (11).	Gent (777.79 ± 366.67), Qi-d (717.86 ± 530.46), Yang-d (588.89 ± 575.42), Phl-d (310.00 ± 490.75), *p* = 0.045	*Gent (64.00 ± 11.69), Qi-d (63.57 ± 15.63), Phl-d (62.78 ± 10.86), Yang-d (58.57 ± 14.09),p = 0.881*	*Gent (70.93 ± 13.94), Qi-d (65.51 ± 22.35), Phl-d (61.19 ± 17.28), Yang-d (57.73 ± 28.01), p = 0.712*	*Phl-d (0.58 ± 0.90), Yang-d (0.56 ± 0.53), Gent (0.44 ± 0.53), Qi-d (0.43 ± 0.51), p = 0.948*	Yang-d (25.00 ± 6.95), Qi-d (18.00 ± 7.99), Phl-d (17.00 ± 5.51), Gent (15.56 ± 4.13), *p* = 0.016
Lu et al. (12)	–	*Phl-d (0.60 ± 0.09), Gent (0.59 ± 0.09), Qi-d (0.55 ± 0.11), Yang-d (0.53 ± 0.10), p = 0.076*	Gent (57.34 ± 11.77), Phl-d (56.32 ± 12.64), Qi-d (48.63 ± 14.02), Yang-d (45.71 ± 10.54), *p* = 0.014	Yang-d (2.32 ± 1.00), Qi-d (2.27 ± 1.26), Phl-d (1.35 ± 0.87), Gent (1.15 ± 0.38), *p* = 0.000	Yang-d(16.11 ± 4.69), Qi-d(13.27 ± 4.75), Phl-d(12.91 ± 4.59), Gent(10.23 ± 3.98), *p* = 0.008
Lin (14)	Yang-d (700.03 ± 302.80), Qi-d (633.01 ± 307.39), Dam-h (344.13 ± 188.03), Blo-s (316.67 ± 183.77), Phl-d (284.29 ± 171.60), *p* < 0.05	–	Dam-h (50.94 ± 12.81), Phl-d (47.14 ± 14.02), Yang-d (40.40 ± 17.23), Qi-d (38.00 ± 17.38), Blo-s (35.83 ± 19.03)*	Yang-d (3.73 ± 1.50), Blo-s (3.67 ± 1.61), Qi-d (3.00 ± 1.65), Dam-h (0.75 ± 0.68), Phl-d (0.43 ± 0.75), *p* < 0.05	–
Gao et al. (15)	–	–	–	Phl-d (3.80 ± 2.353), Qi-d (3.48 ± 2.323), Yin-d (3.22 ± 1.833), Yang-d (2.76 ± 2.215), Qi-dp (2.67 ± 1.751), Blo-s (2.50 ± 1.732), Gent (1.98 ± 1.239), Spe-d (1.83 ± 0.753), *p* = 0.001	–
Liang et al. (16)	–	–	–	–	Qi-d (27.05 ± 6.23), Dam-h (22.77 ± 4.94), Phl-d (18.68 ± 5.41), Yin-d (15.07 ± 3.87), p < 0.05
Wang et al. (17)	–	Yin-d (62.78 ± 15.61), Blo-s (59.05 ± 10.17), Qi-d (51.42 ± 12.69), Yang-d (47.97 ± 10.05), *p* = 0.000	Yin-d (60.83 ± 23.24), Blo-s (52.15 ± 20.73), Qi-d (40.61 ± 17.57), Yang-d (36.05 ± 11.67), *p* = 0.000	Yang-d (2.33 ± 0.63), Qi-d (1.53 ± 0.59), Blo-s (1.43 ± 0.79), Yin-d (0.85 ± 0.66), *p* < 0.05	Yang-d (26.05 ± 4.68), Qi-d (23.15 ± 5.04), Blo-s (20.50 ± 4.35), Yin-d (16.94 ± 5.62), *p* < 0.001
Lu et al. (19)	–	–	Dam-h (66.79 ± 18.25), Qi-dp (65.00 ± 27.12), Blo-s (60.00 ± 22.82), Spe-d (54.00 ± 20.51), Yang-d (53.24 ± 22.69), Phl-d (50.53 ± 25.09), Yin-d (48.33 ± 28.87), Qi-d (46.64 ± 19.02)*	–	–
Wang et al. (20)	–	–	Dam-h (62.18 ± 22.97), Phl-d (53.11 ± 22.64), Qi-d (50.35 ± 23.96), Qi-dp (49.26 ± 25.14), Yin-d (44.86 ± 28.55), Yang-d (30.83 ± 19.51), Blo-s (28.41 ± 14.89)*	–	–
Hou (21)	Qi-d (555 ± 519.63), Blo-s (618 ± 386.84), Yang-d (590 ± 461.89), Yin-d (584.8 ± 483.26), Phl-d (235 ± 526.85), Dam-h (340 ± 519.63)*	–	–	–	–

* The value had been transformed, so the original *p*-value cannot be applied. The italicized data indicates no overall statistical significant differences. Gent: Gentleness, Qi-d: Qi-deficiency, Yang-d: Yang-deficiency, Yin-d: Yin-deficiency, Phl-d: Phlegm-dampness, Dam-h: Dampness-heat, Blo-s: Blood-stasis, Qi-dp: Qi-depression, Spe-d: Specific-diathesis.

### 3.3 Quality assessment and bias risk assessment

Among the eight included cross-sectional studies (9–14, 17–20), four studies were explicitly informed of the quality assurance methods (9–14, 17–20); five studies (9, 10, 12–14, 17, 18) explained why the patients should be excluded from analysis and one study (11) did not; three studies described how confounding was assessed and controlled (11–14). Quality assessment and bias risk assessment are shown in Appendix Figures 1, 2.

Among the three included retrospective or prospective observational studies (15, 16, 21), all three studies got 2 stars in selection section, only one study (21) got 1 star in comparability section, only one study (15) got 3 stars in outcome section. Quality assessment and bias risk assessment are shown in Appendix Figures 3, 4.

### 3.4 Outcomes of direct meta-analysis

The outcomes of direct meta-analysis are shown in Appendix.

### 3.5 Outcomes of network meta-analysis

#### 3.5.1 Smoking index

**Figure 2:**
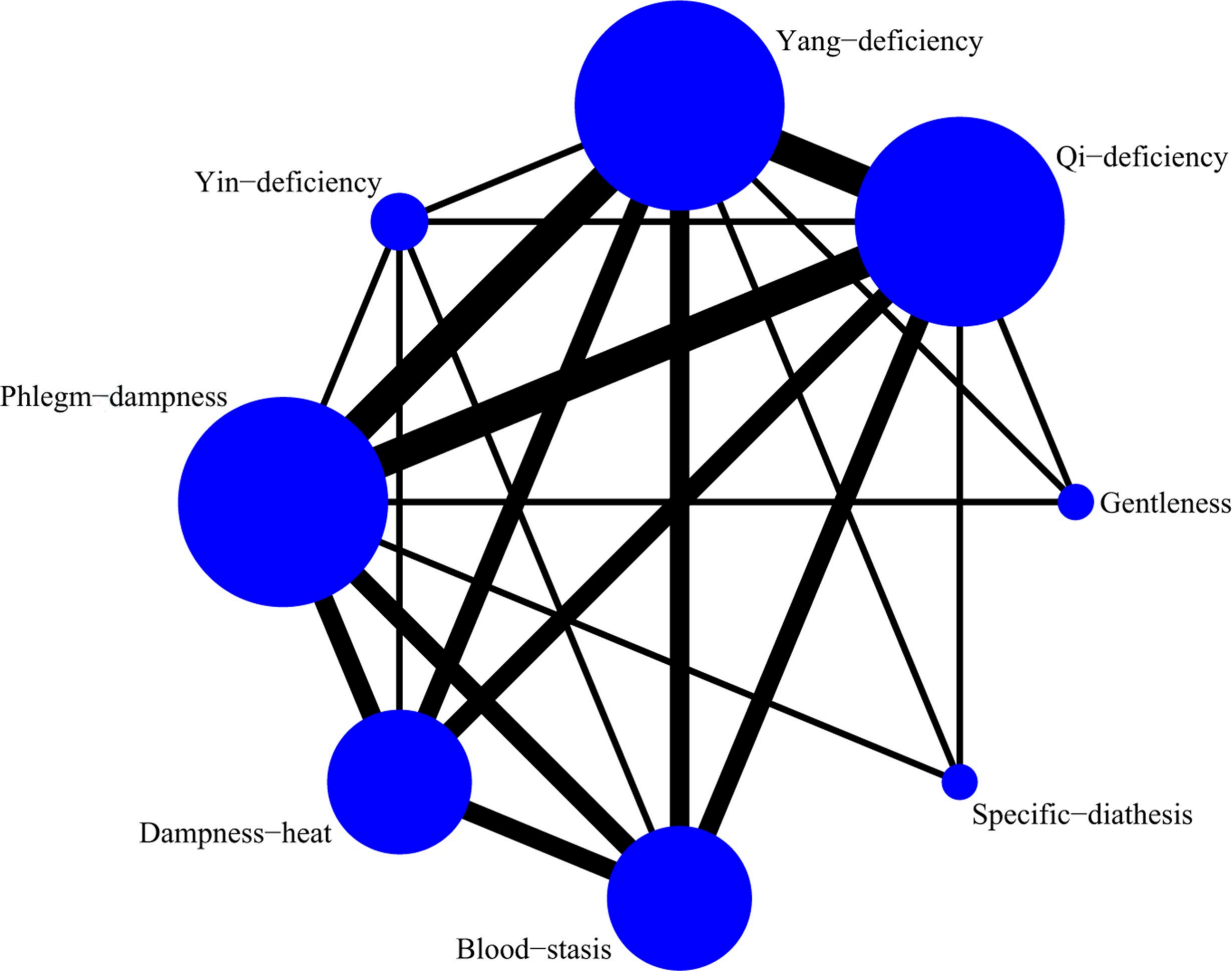
Network plots of smoking index.

**Figure 3:**
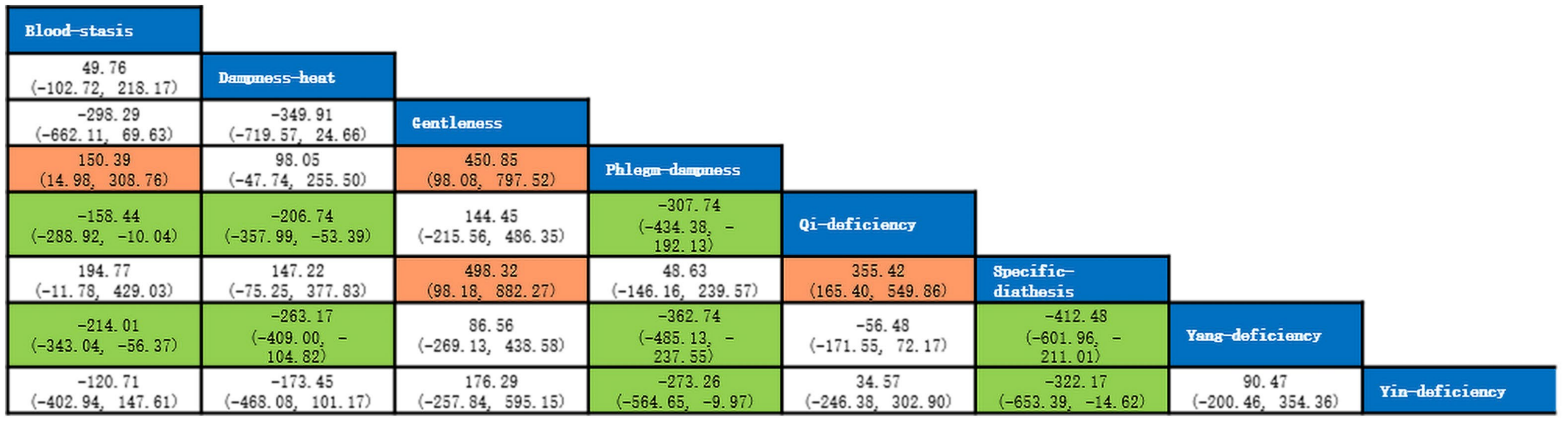
League table of smoking index.

A total of 5 studies with 873 participants were included, and 8 kinds of TCM constitutions with 811 participants were analyzed (9–11, 14, 21). Network plots were shown in Figure 2. After 20,000 turning iterations and 50,000 simulation iterations, the PSRFs did not exceed 1.00. The results of network meta-analysis were shown in Figure 3. As for smoking index of COPD patients, qi-deficiency and yang-deficiency were higher than blood-stasis, dampness-heat, phlegm-dampness and specific-diathesis; gentleness and yin-deficiency was higher than phlegm-dampness and specific-diathesis; blood-stasis was only higher than phlegm-dampness. Based on both the SUCRA (Appendix Figure 10) and the league table, it could be concluded that the smoking index of COPD patients with yang-deficiency, qi-deficiency, gentleness and yin-deficiency were the highest, blood-stasis and dampness-heat were lower, and phlegm-dampness and specific-diathesis were the lowest.

#### 3.5.2 FEV1/FVC

**Figure 4:**
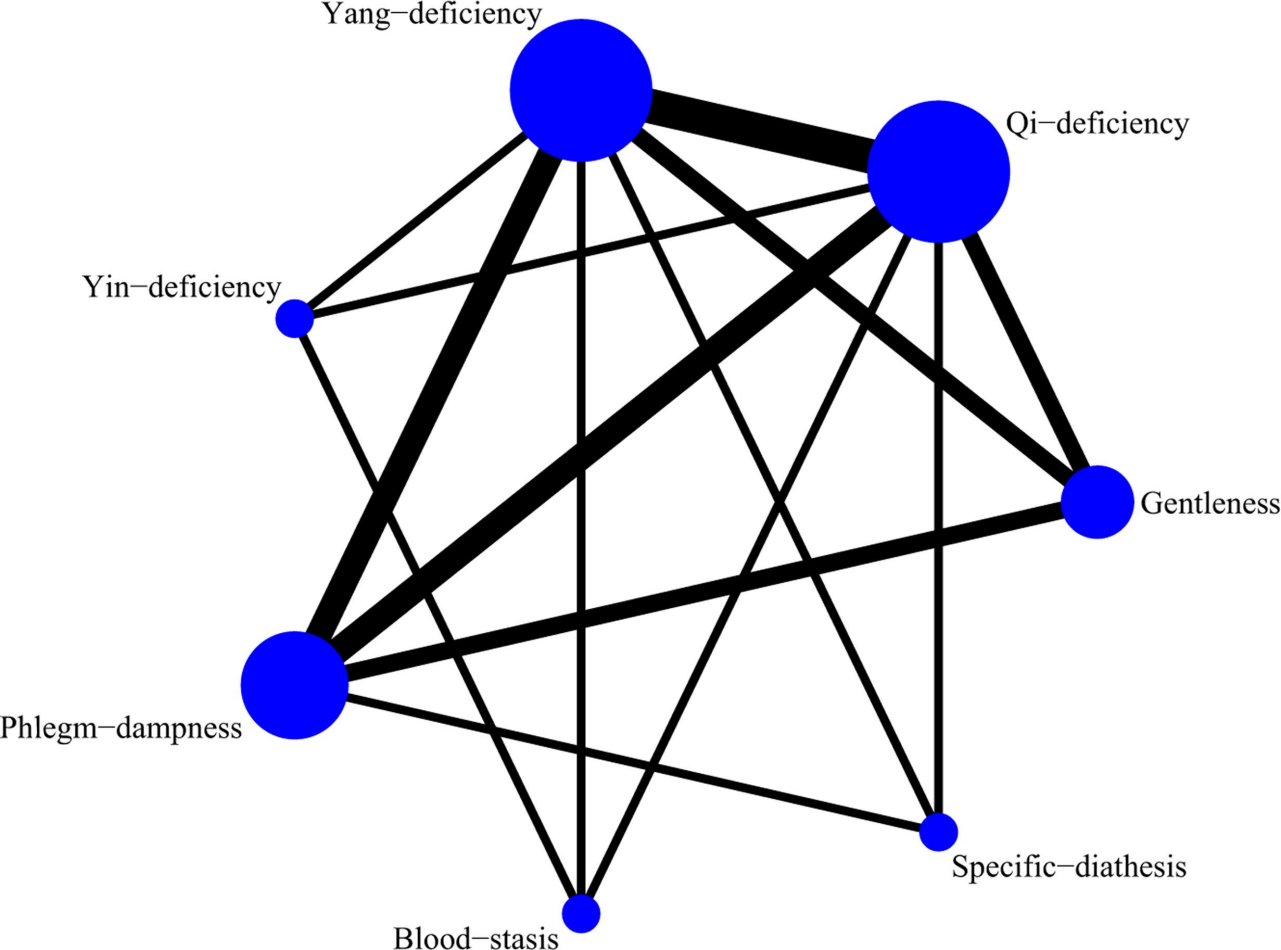
Network plots of FEV1/FVC.

**Figure 5:**
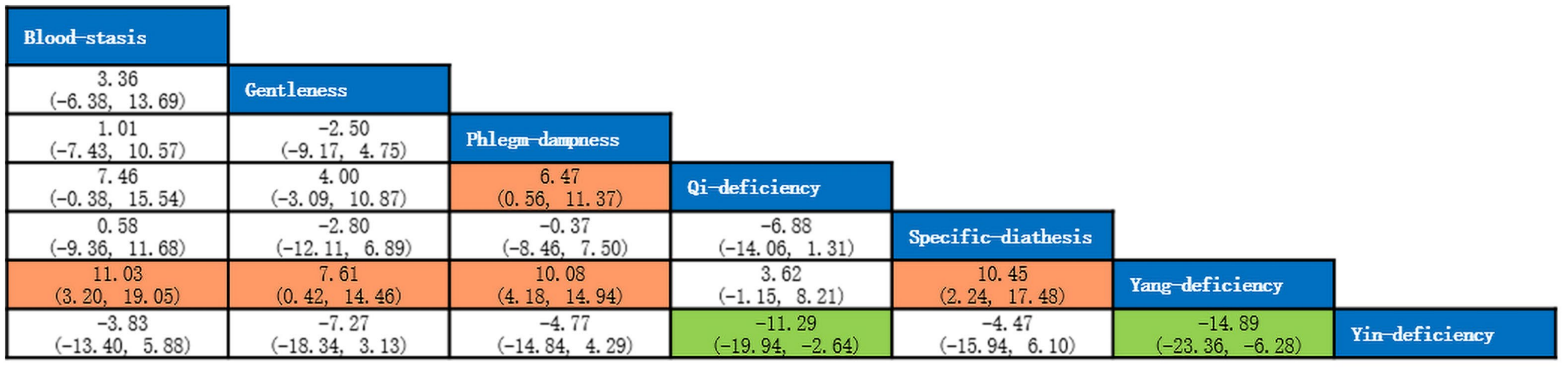
League table of FEV1/FVC.

A total of 4 studies with 641 participants were included, and 7 kinds of TCM constitutions with 600 participants were analyzed (9, 11–13, 17, 18). Network plots were shown in Figure 4. After 20,000 turning iterations and 200,000 simulation iterations, the PSRFs did not exceed 1.01. The results of network meta-analysis were shown in Figure 5. As for FEV1/FVC of COPD patients, yang-deficiency was lower than gentleness, yin-deficiency, phlegm-dampness, blood-stasis and specific-diathesis, and qi-deficiency was lower than yin-deficiency and phlegm-dampness. Based on both the SUCRA (Appendix Figure 11) and the league table, it could be concluded that the FEV1/FVC of COPD patients with yin-deficiency, blood-stasis, specific-diathesis, phlegm-dampness and gentleness were higher, while qi-deficiency and yang-deficiency were lower.

#### 3.5.3 FEV1%Pred

**Figure 6:**
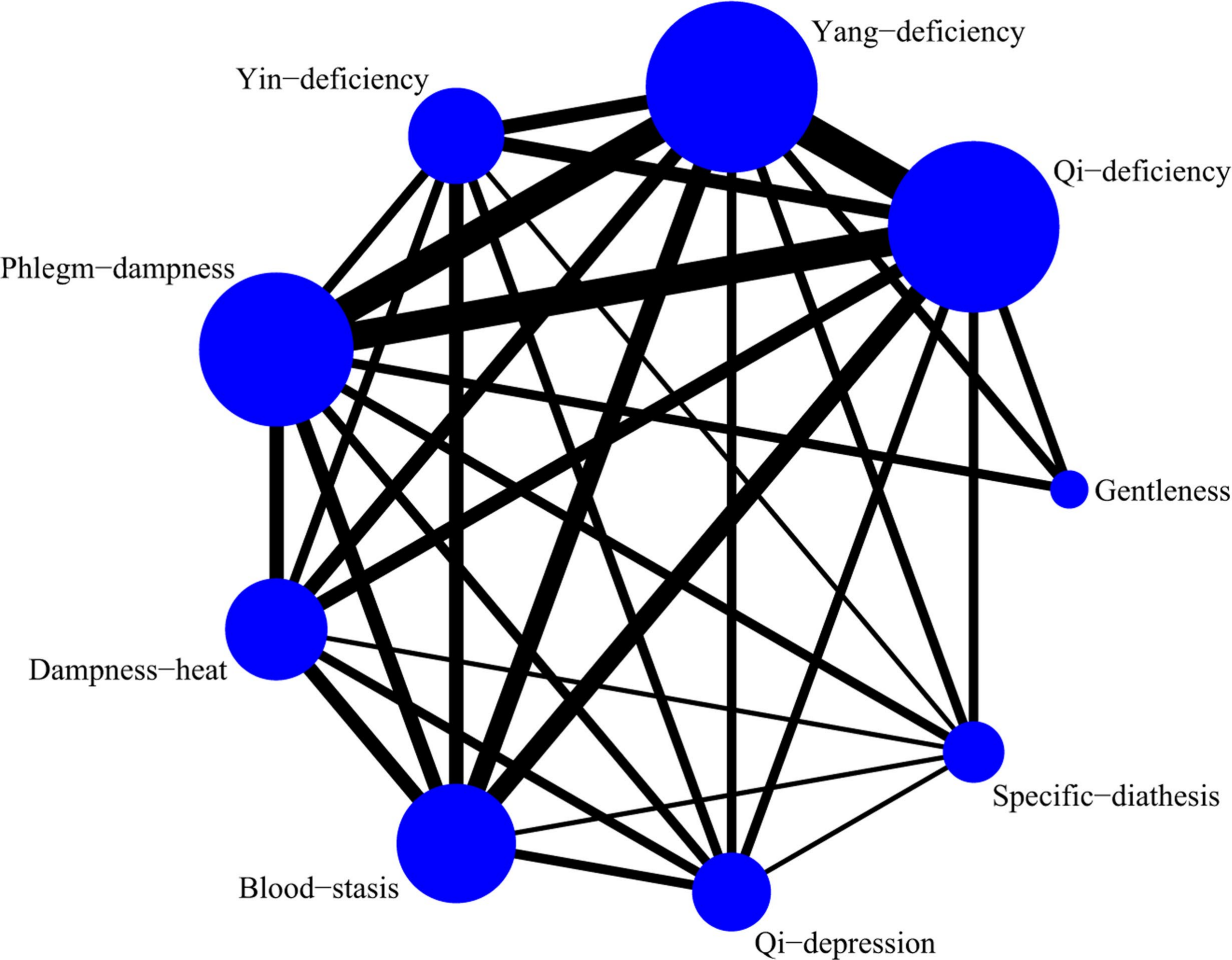
Network plots of FEV1%Pred.

**Figure 7:**
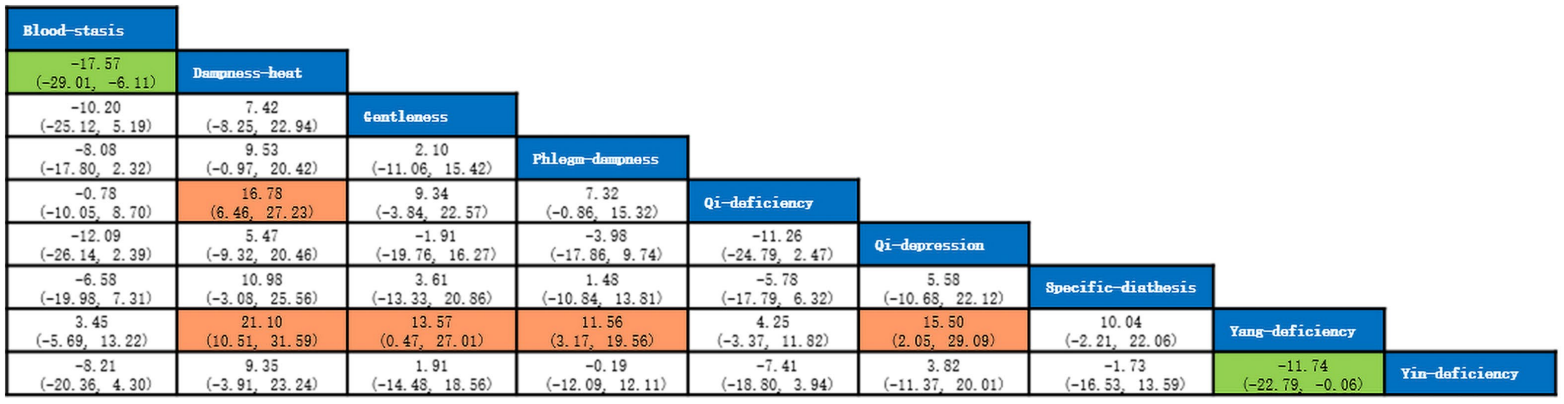
League table of FEV1%Pred.

A total of 7 studies with 1,457 participants were included, and 9 kinds of TCM constitutions with 1,470 person-time were analyzed (In the study of Yingjia Lu et al., each type of the imbalanced constitutions in the composite constitution was analyzed separately, that’s why the number of patients analyzed was more than that of participants) (9, 11–14, 17–20). Network plots were shown in Figure 6. After 20,000 turning iterations and 50,000 simulation iterations, the PSRFs did not exceed 1.00. The results of network meta-analysis were shown in Figure 7. As for FEV1%Pred of COPD patients, yang-deficiency was lower than gentleness, yin-deficiency, phlegm-dampness, dampness-heat and qi-depression, and dampness-heat was higher than qi-deficiency and blood-stasis. Based on both the SUCRA (Appendix Figure 12) and the league table, it could be concluded that the FEV1%Pred of COPD patients with dampness-heat was the highest, qi-depression, gentleness, phlegm-dampness, yin-deficiency and specific-diathesis were lower, qi-deficiency and blood-stasis were much lower, and yang-deficiency was the lowest.

#### 3.5.4 The number of acute exacerbations per year

**Figure 8:**
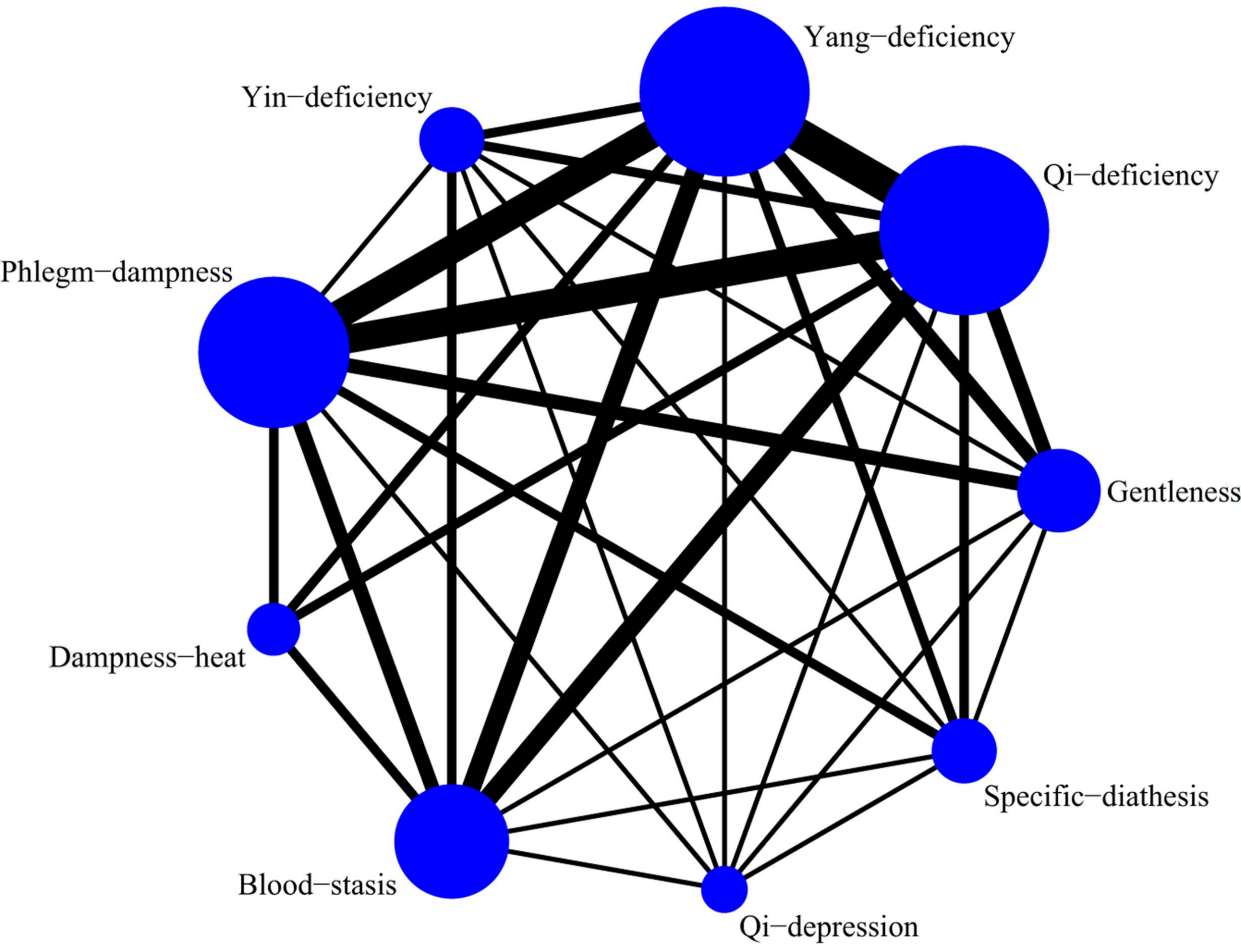
Network plots of AE/y.

**Figure 9:**
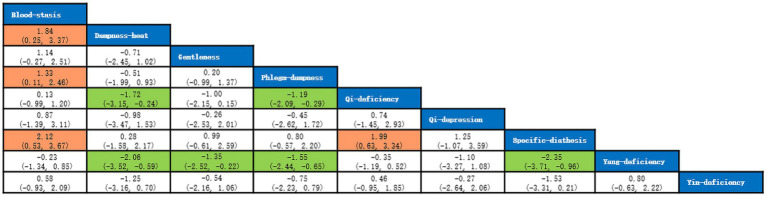
League table of AE/y.

A total of 7 studies with 1,169 participants were included, and 9 kinds of TCM constitutions with 1,188 person-time were analyzed (In the study of Guangfei Gao et al., each type of the imbalanced constitutions in the composite constitution was analyzed separately, that’s why the number of patients analyzed was more than that of participants) (9–15, 17, 18). Network plots were shown in Figure 8. After 20,000 turning iterations and 50,000 simulation iterations, the PSRFs did not exceed 1.00. The results of network meta-analysis were shown in Figure 9. As for AE/y of COPD patients, yang-deficiency, qi-deficiency and blood-stasis were higher than phlegm-dampness, dampness-heat and specific-diathesis, and yang-deficiency was higher than gentleness. Based on both the SUCRA (Appendix Figure 13) and the league table, it could be concluded that the AE/y of COPD patients with yang-deficiency was the highest, blood-stasis and qi-deficiency were lower, yin-deficiency and qi-depression were much lower, and gentleness, phlegm-dampness, dampness-heat and specific-diathesis were the lowest.

#### 3.5.5 CAT score

**Figure 10:**
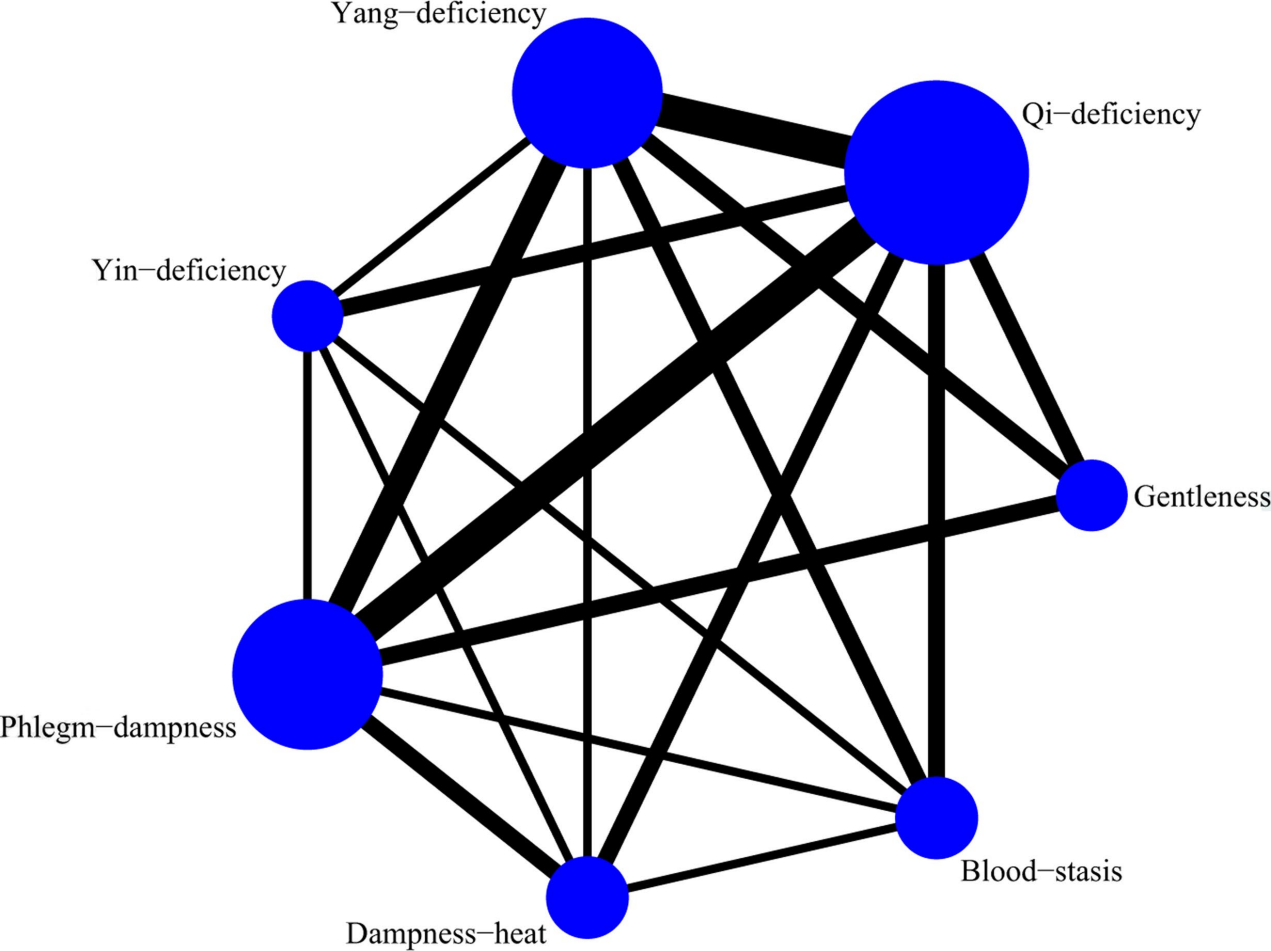
Network plots of CAT score.

**Figure 11:**
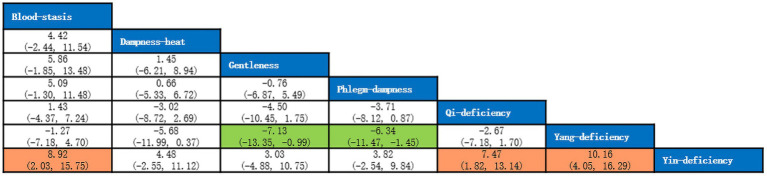
League table of CAT score.

A total of 5 studies with 649 participants were included, and 7 kinds of TCM constitutions with 587 participants were analyzed (10–13, 16–18). Network plots were shown in Figure 10. After 20,000 turning iterations and 50,000 simulation iterations, the PSRFs did not exceed 1.00. The results of network meta-analysis were shown in Figure 11. As for CAT score of COPD patients, yang-deficiency was higher than gentleness, yin-deficiency and phlegm-dampness, and yin-deficiency was lower than qi-deficiency, yang-deficiency and blood-stasis. Based on both the SUCRA (Appendix Figure 14) and the league table, it could be concluded that the CAT score of COPD patients with yang-deficiency, blood-stasis and qi-deficiency were higher, dampness-heat, phlegm-dampness and gentleness were lower, and yin-deficiency was the lowest.

#### 3.5.6 Publication bias

**Figure 12:**
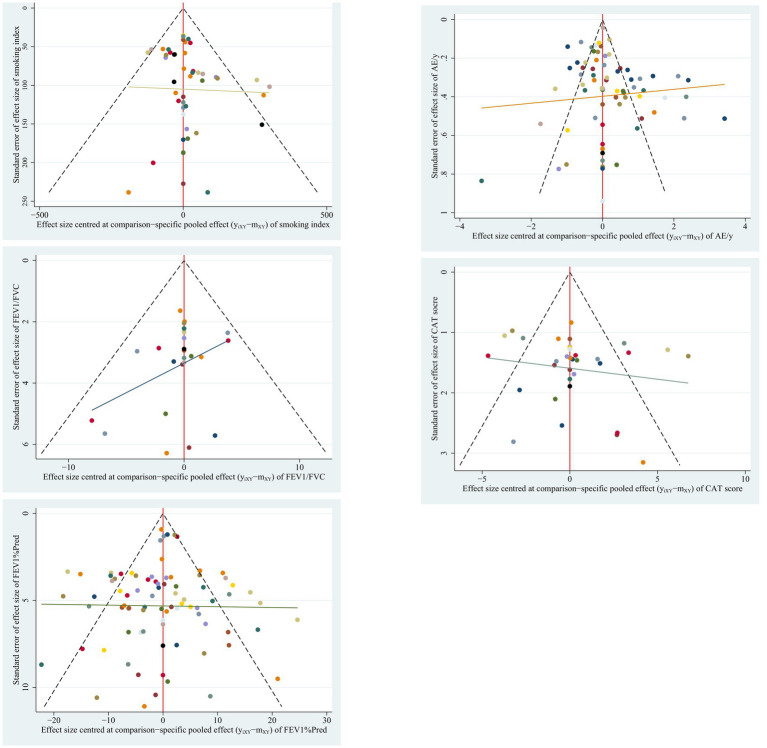
Comparison-adjusted funnel plots of the indexes analyzed.

A comparison-adjusted funnel plot (Figure 12) was drawn for each index to evaluate its potential publication bias. Based on the plot, the distribution of the comparisons of FEV1/FVC was not so symmetrical, and the slope of the fitted line was steep, suggesting a relatively high likelihood of publication bias. Based on network plots, our team believes that this bias came from the limited number of included constitutions and participants in some nodes. Sensitivity analysis can partly resolve this bias by excluding relevant nodes (see 3.5.7). The better solution requires more future research. Conversely, the probability of publication bias of other indexes was relatively low.

#### 3.5.7 Sensitivity analyses

Due to the limited sizes of certain constitutions, the conclusions may be affected. The sensitivity analyses had been conducted to resolve these influences. The two most limited constitutions (qi-depression and specific-diathesis) were excluded from the analyses, and the league tables (Appendix Figures 15–18) revealed unchanged relationships among the rest constitutions, indicating the conclusions were robust.

## 4 Discussion

Based on the above results, we can inferred that the indexes of COPD patients with different TCM constitutions were different. Patients with yang-deficiency demonstrated the poorest outcomes across all indexes, warranting attention from both clinical decision-makers and COPD patients themselves. Patients with other TCM constitutions displayed unique characteristics in different index, hence tailored strategies for prevention, treatment, and rehabilitation should be devised based on individual conditions.

COPD is a chronic respiratory disease with a long history. In the HuangDi neijing, one of the best-known Traditional Chinese Medicine classics, there are disease records as ‘lung-distention disorder’ and ‘dyspnea disorder’, which means ‘deficiency, fullness of chest, cough and wheeze’ and ‘wheeze and nasal flaring’. These diseases have been recently identified as similar to COPD.

TCM constitution is an objectively existing life phenomenon. TCM constitutions can reflect one’s susceptibility to a specific pathogenic factor and the tendency to a specific type of disease. Therefore, by assessing the TCM constitutions, we can predict, to a certain extent, the potential disease tendencies of healthy people and the focus of prevention, treatment, prognosis and rehabilitation of patients. The author will provide an interpretation of TCM constitution in the following aspects.

### 4.1 Classifications and characteristics of TCM constitutions

In general, TCM constitutions can be categorized into 2 main groups: gentleness constitution and imbalanced constitutions. The imbalanced constitutions can further be classified into 8 subcategories. Different imbalanced constitutions can exist independently (simple constitution) or in combination (composite constitution). The detailed classification definitions and characteristics of TCM constitutions are shown in Table 3.

**Table 3:** Classifications and characteristics of TCM constitutions (6, 40).

Content	Gentleness (A)	Qi-deficiency (B)	Yang-deficiency (C)	Yin-deficiency (D)	Phlegm-dampness (E)	Dampness-heat (F)	Blood-stasis (G)	Qi-depression (H)	Specific-diathesis (I)
Definition	Strong and sturdy state with moderate figure, ruddy complexion, and full of energy.	Deficiency of vitality, shortness of breath and low organ and system function.	Deficiency-Cold symptoms caused by deficiency of Yang-Qi.	Deficiency of Yin-Fluid, such as blood and fluid, characterized by inner heat.	Phlegm dampness condenses caused by fluid stasis, characterized by stick and heavy.	Endo-retention of Dampness-Heat.	The potential tendency of poor blood flow and pathological basis of stagnation of blood stasis.	Introverted, unstable, melancholy, vulnerable, sensitive and suspicious caused by Qi stagnation and depression.	Disorders caused by congenital or inherit factors, including allergy diseases, primary immunodeficiency diseases and so on.
General characteristics	Harmony of Yin and Yang, Qi and Blood. Characterized by moderate figure, ruddy complexion, and full of energy.	Deficiency of vitality, characterized by fatigue, shortness of breath, spontaneous sweating and so on.	Deficiency of Yang-Qi, characterized by symptoms of Deficiency-Cold such as fear of cold, cold hands and feet.	Deficiency of Yin-Fluid, characterized by symptoms of Deficiency-Heat such as dry mouth and throat, hot in the central of hands and feet.	Phlegm dampness condenses, characterized by symptoms of Phlegm-Dampness such as fat figure, abdominal obesity, sticky mouth and tongue.	Endo-retention of Dampness-Heat, characterized by symptoms of Dampness-Heat such as greasy face, bitter mouth, yellow and greasy tongue-fur.	Blood flow is not smooth, characterized by symptoms of Blood-Stasis such as dark skin or dark-purple tongue.	Qi stagnation, characterized by symptoms of Qi-Depression such as depression, anxiety and fragility.	Congenital disorders, characterized by physiological defects, allergic reactions and so on.
Body structure characteristics	Finely developed and strong	Floppy and unsolid muscles	Floppy and unsolid muscles	Thinnish	Fat figure, abdominal obesity	Moderate or thinnish figure	Fat or thin	Mainly thin	Nothing special for people with allergic constitution, and people with congenital disorders may have malformations or physiological defects.
Mental characteristics	Easygoing and bright	Introverted and unadventurous	Quiet and introverted	Irritable, outgoing and active	Gentle, steady and patience	Easily upset or irritable	Easily bothered and/or forgetful	Introverted, unstable and sensitive	Varies
Disease tendency	Have a relatively low risk to diseases.	Susceptible to colds, visceroptosis and so on, and recovery slowly.	Susceptible to Phlegm-fluid, swelling, diarrhea and so on. Easily attacked by Pathogenic-Cold.	Susceptible to fatigue, spermatorrhea, insomnia and so on. Easily attacked by Pathogenic-Heat.	Susceptible to diabetes mellitus, stroke and coronary heart disease.	Susceptible to furunculosis, jaundice, urinary tract infections and so on.	Susceptible to Gynecologic Abdominal Lumps, pain syndrome, blood syndrome and so on.	Susceptible to hysteria, globus hystericus, lily disease and depression.	People with allergic constitution are susceptible to asthma, urticaria, hay fever and medicine allergy, etc.; with hereditary diseases are susceptible to hemophilia, down’s syndrome, etc.; with fetal diseases are susceptible to five kinds of retardations/flaccidity, non-closure of fontanel, Infantile epilepsy, etc.
Environmental adaptability	Strong adaptability to natural and social environment.	Intolerance to pathogenic Wind, Cold, Heat and Dampness.	Tolerance to summer but not winter, susceptible to pathogenic Wind, Cold and Dampness.	Tolerance to winter but not summer, susceptible to pathogenic Summer, Heat and Dry.	Intolerance to plum rain season and very wet environment.	Intolerance to the damp-heat climate at the end of summer or the beginning of fall, very wet or hot environment.	Intolerance to pathogenic Cold.	Intolerance to mental stimulation and wet or rainy weather.	Poor adaptability, e.g., people with allergic constitution are poorly adapted to seasons of allergy and are easily sick.

### 4.2 Relationship between TCM constitution, body constitution and phenotype

TCM constitution differs from body constitution in modern medicine. Body constitution refers to the physical characteristics of the body, encompassing the mode of performance of functions, the activity of metabolic processes, the manner and degree of reactions to stimuli, and power of resistance to the attack of pathogenic organisms. It includes indexes such as body height, body weight, body fat distribution, body surface area, etc. On the other hand, TCM constitution is more like phenotype, which refers to the outward appearance of the individual. Phenotype is the product of interactions between genes, and between the genotype and the environment. In modern medicine, different diseases have their own classifications of phenotype. A COPD phenotype is a single or combination of disease attributes that describe differences between individuals with COPD as they relate to clinically meaningful outcomes (symptoms, exacerbations, response to therapy, rate of disease progression, or death) (22). The classic phenotypes of COPD include asthma, emphysema, chronic bronchitis, and so on. However, unlike phenotype, TCM constitution can be applied to both healthy and ill people, and can be used for evaluation and treatment under specific diseases together with Traditional Chinese Medicine symptom (TCM symptom).

### 4.3 Proofs of TCM constitution in modern medicine

The TCM constitution theory originated from Huangdi Neijing, and finally established the nine-class classification system in this century after a long period of development. Numerous modern studies have provided support for the TCM constitution theory in various aspects. In genomics, Cao et al. found that abnormal mutations in the corresponding loci of the ZYG11A and LOXL2 genes were detected in all three generations of the yin-deficiency family, indicating a familial predisposition; studies of Dong et al. and Wang et al. also identified associations between genomics and TCM constitution (23–26). In metabolomics, Liu et al. found that the overall energy metabolism level of patients with yang-deficiency was low and their lipid and glucose metabolism were in disorder by comparing the plasma metabolites of patients with gentleness and yang-deficiency; other relationships of metabolomics and TCM constitution had been revealed by studies of Liu et al. (27), Chai et al., (28), Wang et al. (29), and Li (30). Therefore, the TCM constitution theory is not only an accumulation of Traditional Chinese Medicine, but also a crystallization of modern medical research.

### 4.4 Relationship between TCM constitution and indexes in COPD patients

#### 4.4.1 Smoking index

In COPD patients, the smoking index of patients with yang-deficiency and qi-deficiency was relatively high. According to the Southern Yunnan herb, tobacco is considered as a “poison” of bitter-warm, which easily generates pathological products of internal-heat and phlegm-dampness, and prolonged smoking will deplete yang-qi and lead to a constitution of deficiency, leading to common occurrences of yang-deficiency and qi-deficiency in patients with high smoking indices. This conclusion is partly consistent with the result of Qiao (9, 31). Contrary to initial assumptions, patients with gentleness demonstrated a high smoking index. As mentioned above, individuals with gentleness constitution have a relatively low risk to diseases; therefore, if they do get sick, there should be strong pathogenic factors. In this study, long-term heavy smoking was considered one such strong pathogenic factor.

#### 4.4.2 Lung-function indexes (FEV1/FVC and FEV1%Pred)

In COPD patients, the FEV1/FVC and FEV1%Pred of patients with yang-deficiency and qi-deficiency were relatively low (patients with yang-deficiency were lower), while FEV1%Pred of patients with blood-stasis were lower than qi-deficiency. According to Traditional Chinese Medicine, COPD belongs to the category of “lung-distention,” and its pathological nature is “root deficiency and branch excess.” In the early stages, “excess-pathogen” could be seen in most patients, which mainly consist of “Turbid-phlegm” and “Fluid-retention.” As the disease progresses, vital-qi becomes depleted. Since the lung connects all vessels and heart qi is connected with the lung, pulmonary disease could injury the heart gradually. With declining heart qi and yang, the constitution gradually shifts to deficiency or stasis, presenting symptoms such as wheezing, low voice, shortness of breath, fear of cold and cold limbs. These symptoms are also the characteristics of qi-deficiency and yang-deficiency. Simultaneously, the lung function also gradually declines as the disease progresses. As a result, patients with qi-deficiency and yang-deficiency tend to have poorer lung function. In the advanced stages, COPD patients often develop chronic hypoxia and cor pulmonale, which can easily lead to hemodynamic abnormalities and coagulation activation, and blood-stasis formed. Therefore, patients with blood-stasis also exhibit poor lung function (6, 9, 14). This result is consistent with Lu et al.’s study on the correlation between TCM constitution and COPD grading (19). Studies of Xu et al. and Xueqing Ye et al. revealed that patients with yin-deficiency exhibited hyperactivated sympathetic nerve function, while those with yang-deficiency experienced increased parasympathetic nerve function and decreased sympathetic nerve function (32, 33). Considering the role of sympathetic nerves in airway smooth muscle relaxation and the opposite effect of parasympathetic nerves, these studies may offer a potential explanation for our findings.

#### 4.4.3 The number of acute exacerbations per year

In COPD patients, AE/y of patients with yang-deficiency was the highest, followed by blood-stasis and qi-deficiency. According to the study of Xu et al., the cellular immune function of patients with qi-deficiency in lung and kidney and yang-deficiency was low, while it was normal or relatively normal in patients with “excess pathogen,” indicating that normal immune function was associated with sufficient vital-qi. Tong Wu et al. found that compared to patients with gentleness constitution, patients with qi-deficiency and yang-deficiency had lower levels of CD4, CD8 and CD4/CD8, which was an indication of immune dysfunction. Additionally, Qi Wang et al. discovered that the HPA axis and Hypothalamus-Pituitary-Thyroid (HPT) axis function of people with yang-deficiency were declined, and their cyclic adenosine monophosphate (cAMP) system and immune function were in disorder (11, 29, 34). Furthermore, another study of Wang et al. revealed that patients with yang-deficiency exhibited elevated level of IL-1β, compared to those with gentleness constitution (29). It is well-established that higher IL-1β level is associated with increased exacerbation rate and decreased FEV1/FVC and FEV1%Pred. In Traditional Chinese Medicine, patients with qi-deficiency were insufficiency of lung qi and weak in external-defense, making them susceptible to exogenous pathogen invasion; patients with yang-deficiency lack warmth and are susceptible to pathogenic cold; the lung is fragile and close to the surface, leading it to be easily invaded if the pathogenic-qi enters through the mouth and nose; hence COPD patients with qi-deficiency and yang-deficiency are more susceptible to acute exacerbations. In modern medicine, the GOLD guidelines also point out that exacerbations are mainly triggered by respiratory infections. And as mentioned above, one of the characteristics of patients with yang-deficiency and qi-deficiency is immune dysfunction, which renders them susceptible to lung infections and COPD exacerbations. The study of Xu et al. found that as blood-stasis progresses, a hypercoagulable state tended to be formed, leading to thrombosis and disease progression (35). The GOLD guidelines also stated that an increased ratio of the pulmonary artery to aortic cross sectional dimension was associated with acute exacerbation of COPD. These could provide potential explanations for the high AE/y in patients with blood-stasis.

#### 4.4.4 CAT score

The CAT questionnaire is a comprehensive health status assessment for COPD patients. Patients with yang-deficiency, blood-stasis and qi-deficiency achieved higher CAT scores. The primary symptoms of COPD include cough, phlegm production and wheezing. As previously mentioned, in Traditional Chinese Medicine, the lung is susceptible to pathogenic-qi invasion, resulting in its dysfunction, then the lung-qi reverse upwardly to form coughing. With disease progression, the spleen and kidney are affected by lung disease, leading these three zang viscera to a status of deficiency. Because of the dysfunction of spleen in transportation, the phlegm-dampness is generated in the body, then it moves to the lung and the phlegm is formed. Since the lung is the master of qi and the kidney is the root of qi, lung cannot master qi and kidney cannot receive qi when patients are in the status of lung-kidney deficiency, causing worsening wheezing symptoms. As the disease advances, the lung disease would affect the heart, and the deficiency of qi would affect yang, making it difficult to warm and promote blood, then yin, yang, qi and blood are all in disorder, and more symptoms, insomnia due to mental disorder, for example, occur. Therefore, when the TCM constitution shifts toward deficiency or stasis, the cardiopulmonary function tends to deteriorate and symptoms worsen. Additionally, there are notable similarities between the 8-items of CAT assessment and the 8-items of qi-deficiency judgment. These could potentially elucidate why the CAT scores were higher in patients with yang-deficiency, blood-stasis and qi-deficiency.

As previously mentioned, COPD patients with yang-deficiency and qi-deficiency exhibit poorer outcomes across all indexes. Therefore, for clinical decision-makers and practitioners, it is important to support vital-qi, warm and activate yang, and tonify qi based on TCM syndrome differentiation during the treatment of COPD patients. Patients with blood-stasis perform poorly in FEV1%Pred, AE/y and CAT score. Hence, treatment should focus on promoting blood circulation to alleviate stasis and addressing small airway resistance, thrombosis, cardiac function as well as activity tolerance. Moreover, if other TCM constitutions, such as phlegm-dampness, dampness-heat, Yin-deficiency, etc., tend to shift to the aforementioned constitutions, it indicates that the patient may be at risk of disease progression and should be given additional attention. Treatment and rehabilitation strategies should be tailored according to the specific TCM constitutions, e.g., smoking cessation should be more emphasized in patients with gentleness constitution. The framework and direction of the interventions were concluded (Appendix Table 2) based on the conclusions of this study, and the actionable methods are referred to the relevant literature (1, 36).

There are several limitations in this study. Firstly, the majority of participants included were from the southeast and northeast regions of China, which lacks representation from southwest and northwest regions. Given that TCM constitution could be influenced by the environment, there is a potential risk of bias. However, this risk is limited because: 1. The environment influences mainly on the distribution of the constitution, not the relationship discussed; 2. The population of this study represents the region below the Heihe-Tengchong line, the region contains 93% of China’s population, making a good population representation. Secondly, indexes other than FEV1%Pred and AE/y did not encompass all 9 types of TCM constitutions in this study. In the original studies, some specific TCM constitutions were excluded from analysis due to their too small sample size. This made the overall conclusion incomplete, and may introduce risk of bias. Lastly, although this study included 11 studies with a total of 2,168 participants, some indexes had relatively small sample sizes, with the smallest being only 641 participants, thus increasing the likelihood of bias.

Given the large number of comparison groups (9 TCM constitutions), NMA was selected as the statistical method. However, applying NMA to observational studies entails certain risks, including increased confounding bias, insufficient power for inconsistency testing, and low reliability of ranking results. For example, although the included studies were generally comparable with respect to age distribution, substantial variation in sex distribution was observed across studies, which was arising from the known sex imbalance in the COPD population, and this variation could introduce confounding bias. We attempted to adjust for this potential confounding using network meta-regression. However, the model did not converge. Therefore, future studies with more original individual-level data per study arm are needed. Furthermore, heterogeneity in follow-up durations across studies contributes to increased variability in effect estimates, which may reduce the statistical power of inconsistency tests. Therefore, the findings of this study indicate only correlational relationships between TCM constitutions and the aforementioned COPD indicators, and do not imply causality. Nevertheless, the risk of inconsistency was mitigated by the fact that all studies included in this study were multi-arm studies, which inherently strengthen the network connectivity and reduce reliance on indirect comparisons. Furthermore, the consistency between the NMA results and pair-wise meta-analyses supports the relative robustness of our conclusions.

There are some aspects we think could be investigated on the relationship between TCM constitution and indexes in COPD patients in future studies. The outcome of AE/y in this study was consistent with most studies. However, different outcomes have been reported in other studies and the outcomes were somewhat consistent with each other (the AE/y of patients with phlegm-dampness was higher than other TCM constitutions) (11, 15, 37, 38). To address this discrepancy, potential solutions could involve modifying certain conditions, such as increasing sample size, establishing subgroups or adjusting baseline characteristics. Furthermore, it is worth noting that previous studies, including this one, have primarily focused on simple constitutions. However, in reality, multiple imbalanced constitutions may coexist in one person (known as composite constitution), and mixed or extra characteristics may be presented. Therefore, investigating composite constitutions could be a promising direction for future research. Lastly, recent studies have emphsized on the relationship between COPD exacerbations and biomarkers, which are ideal tools for linking phenotypes to endotypes (39). Unfortunately, there is no clear study on the difference of biomarkers between COPD patients with different TCM constitutions. This could also be a valuable future research direction.

In conclusion, the indexes of COPD patients with different TCM constitutions are varied. Patients with yang-deficiency and qi-deficiency exhibit poor performance in all indexes, while patients with blood-stasis are poor in all indexes except smoking index. Patients with other TCM constitutions demonstrate distinct characteristics. Although the above conclusions indicate a correlational rather than a causal association, these characteristics nonetheless merit clinicians’ attention in decisions regarding treatment, prognosis, and rehabilitation.

## Data Availability

The original contributions presented in the study are included in the article/Supplementary material, further inquiries can be directed to the corresponding author/s.
