# Supplementary material for: The relationship between traditional Chinese medicine constitution and indexes in chronic obstructive pulmonary disease patients: a systematic review and network meta-analysis
Source: Front Med (Lausanne). 2026 May 22;13:1815235. doi: 10.3389/fmed.2026.1815235 (PMC13237687; doi:10.3389/fmed.2026.1815235)
Supplement: Supplementary file 1 [file Data_Sheet_1.docx]

The relationship between Traditional Chinese Medicine constitution and indexes in chronic obstructive pulmonary disease patients:

A systematic review and network Meta-analysis

**Sheng Xie^1^, Meiling Xie^2^, Guanhong Li^1^, Yu Zhang^1^, Hui Wang^1^, Yuqiong Zheng^1^**

**Appendix**

_______________________________________________________________________________

1. Department of Pulmonary and Critical Care Medicine, Chengdu first People’s Hospital, Sichuan, China

2. Department of Traditional Chinese Medicine, Sichuan Electric Power Hospital, Sichuan, China

Communication author: Yuqiong Zheng, Department of Pulmonary and Critical Care Medicine, Chengdu first People’s Hospital, Sichuan, China. Email: 820523637@qq.com

1. **Search strategy**

We searched online academic platforms from inception to March 31th, 2026. The platforms included Cochrane Library, Embase (using Ovid platform), PubMed in English and CNKI, Wangfang database, VIP and SinoMed in Chinese. Based on the PICOS framework (see 2.4 Selection criteria), we selected the following MeSH terms: “Pulmonary Disease, Chronic Obstructive”, “Medicine, Chinese Traditional”, “肺疾病,慢性阻塞性”, “中医学”. Search keywords were generated accordingly, and pilot searches were performed to determine the optimal keywords and Boolean search strings. Retrieved records were exported to Endnote. After duplicate removal via both automated and manual checks, the remaining articles were screened and discussed according to the selection criteria. The final list of included studies was thus determined. Detailed information on the search platforms and the corresponding Boolean search strings is shown in Appendix Table. 1.

| Academic platform | Boolean search strings |
| --- | --- |
| Pubmed | ("chronic obstructive pulmonary disease" OR COPD) AND ("body constitution of TCM" OR "body constitution of traditional Chinese medicine" OR "TCM body constitution" OR "traditional Chinese medicine body constitution" OR "constitution of TCM" OR "constitution of traditional Chinese medicine" OR "TCM constitution" OR "traditional Chinese medicine constitution" OR "constitution of CM" OR "constitution of chinese medicine" OR "chinese medicine constitution" OR "CM constitution") |
| Embase | ("chronic obstructive pulmonary disease" OR COPD) AND ("body constitution of TCM" OR "body constitution of traditional Chinese medicine" OR "TCM body constitution" OR "traditional Chinese medicine body constitution" OR "constitution of TCM" OR "constitution of traditional Chinese medicine" OR "TCM constitution" OR "traditional Chinese medicine constitution" OR "constitution of CM" OR "constitution of chinese medicine" OR "chinese medicine constitution" OR "CM constitution") |
| Cochrane library | ("chronic obstructive pulmonary disease" or "COPD" in Title Abstract Keyword) AND ("body constitution of TCM" OR "body constitution of traditional Chinese medicine" OR "TCM body constitution" OR "traditional Chinese medicine body constitution" OR "constitution of TCM" OR "constitution of traditional Chinese medicine" OR "TCM constitution" OR "traditional Chinese medicine constitution" OR "constitution of CM" OR "constitution of chinese medicine" OR "chinese medicine constitution" OR "CM constitution" in Title Abstract Keyword) |
| CNKI | (慢性阻塞性肺疾病 + 慢阻肺 + 慢性阻塞性肺病 + 慢性阻塞性肺部疾病) * 中医体质 |
| Wanfang database | 主题:(慢性阻塞性肺疾病 or 慢阻肺 or 慢性阻塞性肺病 or 慢性阻塞性肺部疾病) and 主题:(中医体质) |
| VIP | (慢性阻塞性肺疾病 or 慢性阻塞性肺部疾病 or 慢性阻塞性肺病 or 慢阻肺) and 中医体质 |
| Sinomed | (U=慢性阻塞性肺部疾病 or U=慢性阻塞性肺疾病 or U=慢性阻塞性肺病 or U=慢阻肺) and U=中医体质 |

Appendix Table. 1 The academic platforms and the corresponding Boolean search strings.

**2. Quality assessment and bias risk assessment**

Appendix Fig. 1 Quality assessment of included cross-sectional studies. The details of items 1-11 were shown in 2.6 of the main manuscript.


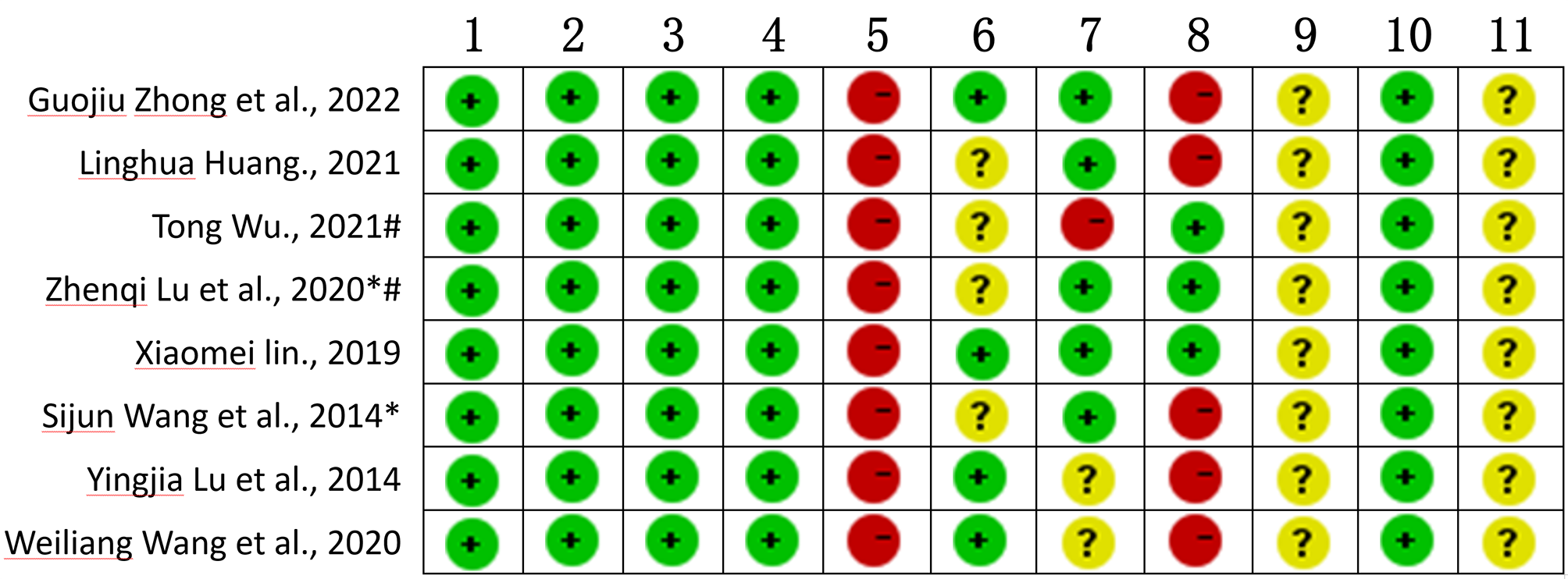


Appendix Fig. 2 Bias risk assessment of included cross-sectional studies. The details of items 1-11 were shown in 2.6 of the main manuscript.

Appendix Fig. 3 Quality assessment of included retrospective or prospective observational studies. The details of items 1-8 were shown in 2.6 of the main manuscript.


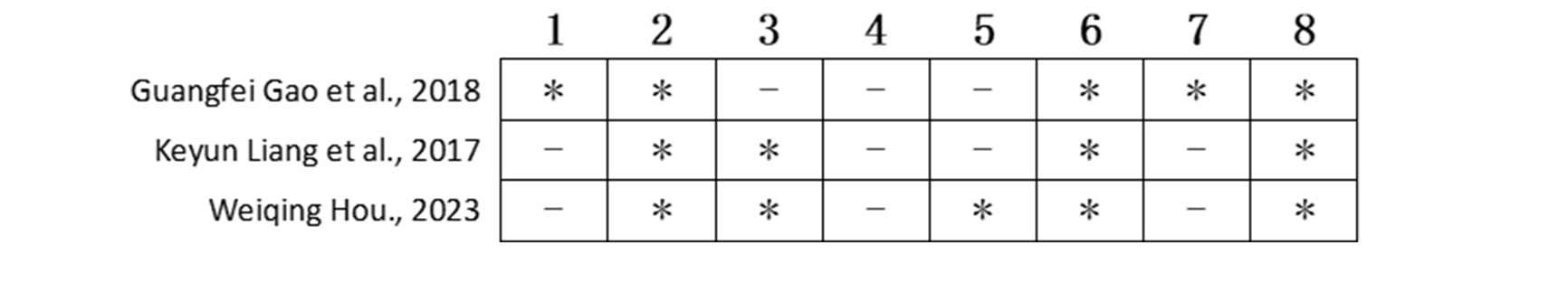


Appendix Fig. 4 Bias risk assessment of included retrospective or prospective observational studies. The details of items 1-8 were shown in 2.6 of the main manuscript.

**3.Outcomes of direct meta-analysis**

**3.1 Smoking index**

A total of 5 studies with 873 participants were included, and 8 kinds of TCM constitutions with 811 participants were analyzed ^[10-12,15,39]^. As shown in Apendix Fig. 3, compared to gentleness constitution, the smoking index of phlegm-dampness, dampness-heat and specific-diathesis was lower with statistical significance (p<0.05). Among the imbalanced constitutions, the smoking index of qi-deficiency and yang-deficiency was higher than other constitutions except yin-deficiency, and that of yin-deficiency and blood-stasis was higher than phlegm-dampness and specific-diathesis. There was no statistical difference between other constitutions (p≥0.05).

Appendix Fig. 5 Comparison of smoking index between TCM constitutions.

**3.2 FEV1/FVC**

A total of 4 studies with 641 participants were included, and 7 kinds of TCM constitutions with 600 participants were analyzed ^[9,11-13,17,18]^. As shown in Apendix Fig. 4, compared to gentleness constitution, only the FEV1/FVC of yang-deficiency was lower with statistical significance (p<0.05). Between the imbalanced constitutions, the FEV1/FVC of patients with yang-deficiency was lower than other constitutions, qi-deficiency was lower than other constitutions except yang-deficiency. There was no statistical difference between other constitutions (p≥0.05).


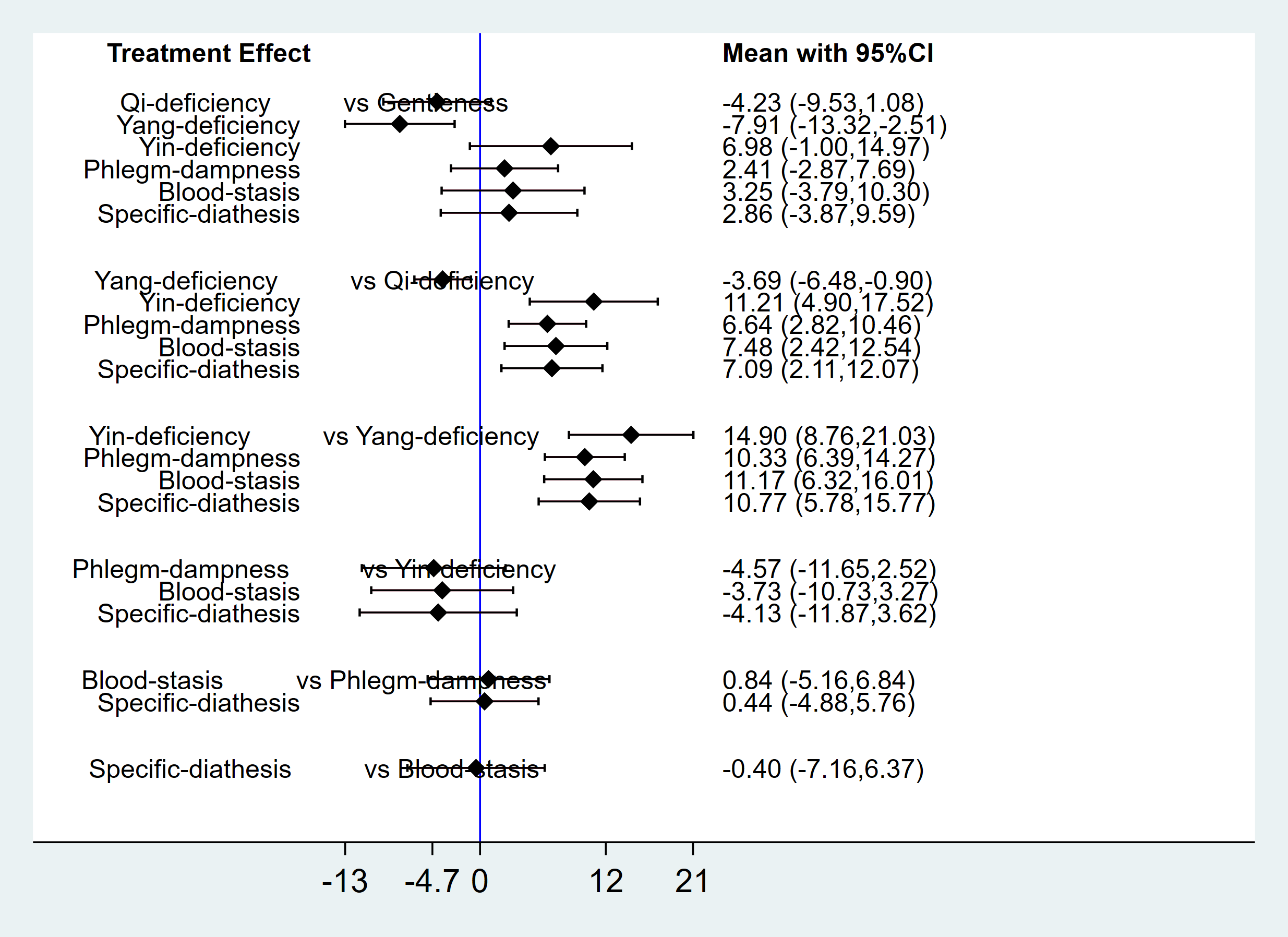


Appendix Fig. 6 Comparison of FEV1/FVC between TCM constitutions.

**3.3 FEV1%Pred**

A total of 7 studies with 1457 participants were included, and 9 kinds of TCM constitutions with 1470 person-time were analyzed ^[9,11-14,17-20]^. As shown in Apendix Fig. 5, compared to gentleness constitution, only the FEV1%Pred of yang-deficiency was lower with statistical significance (p<0.05). Between the imbalanced constitutions, the FEV1%Pred of patients with yang-deficiency was lower than yin-deficiency, phlegm-dampness, dampness-heat and qi-depression, dampness-heat was higher than qi-deficiency, yang-deficiency and blood-stasis. There was no statistical difference between other constitutions (p≥0.05).

Appendix Fig. 7 Comparison of FEV1%Pred between TCM constitutions.


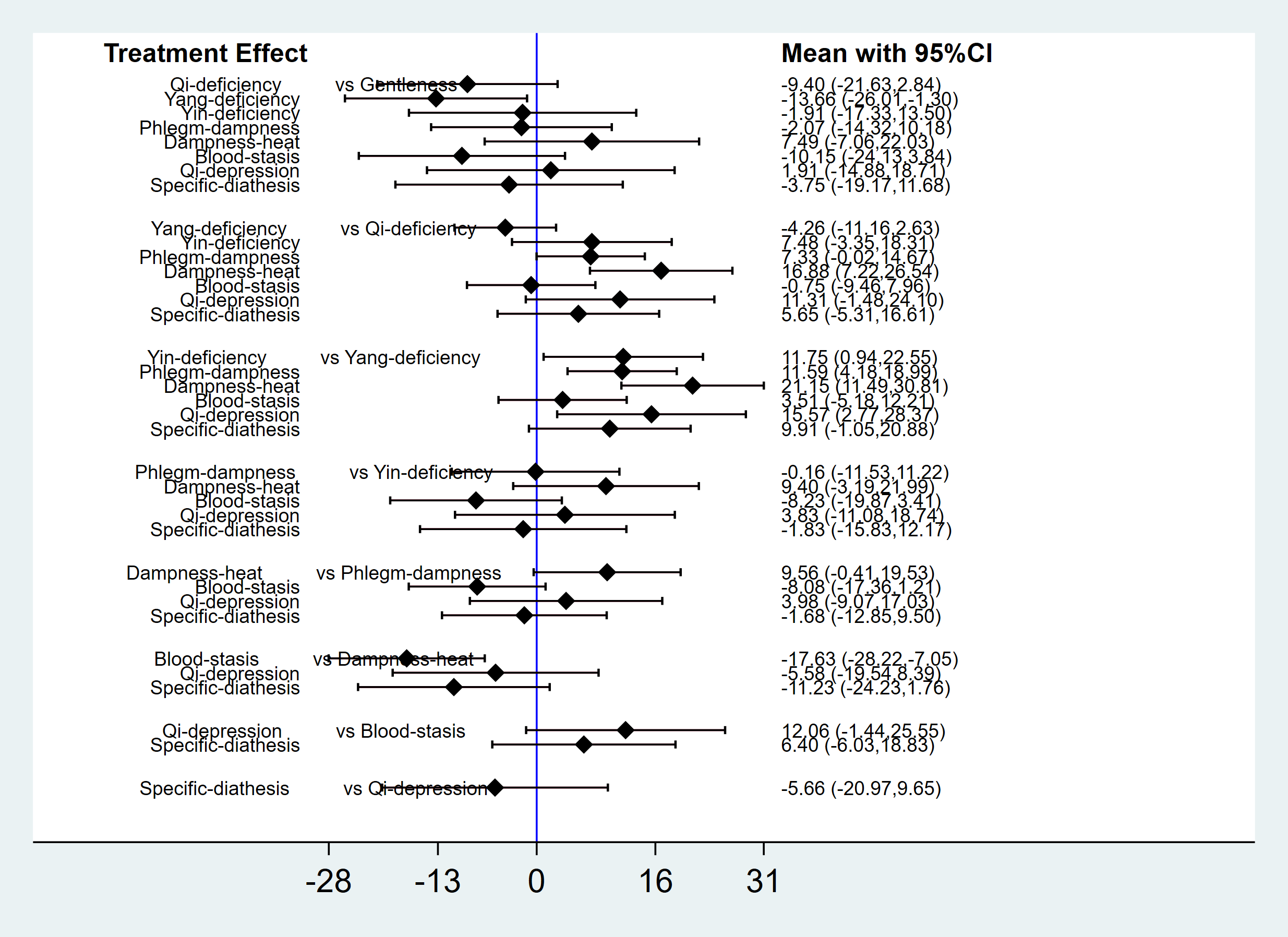


**3.4 The number of acute exacerbations per year (AE/y)**

A total of 7 studies with 1169 participants were included, and 9 kinds of TCM constitutions with 1188 person-time were analyzed. As shown in Apendix Fig. 6, compared to gentleness constitution, only the AE/y of yang-deficiency was higher with statistical significance (p<0.05). Between the imbalanced constitutions, the AE/y of patients with qi-deficiency, yang-deficiency and blood-stasis were higher than phlegm-dampness, dampness-heat and specific-diathesis. There was no statistical difference between other constitutions (p≥0.05).


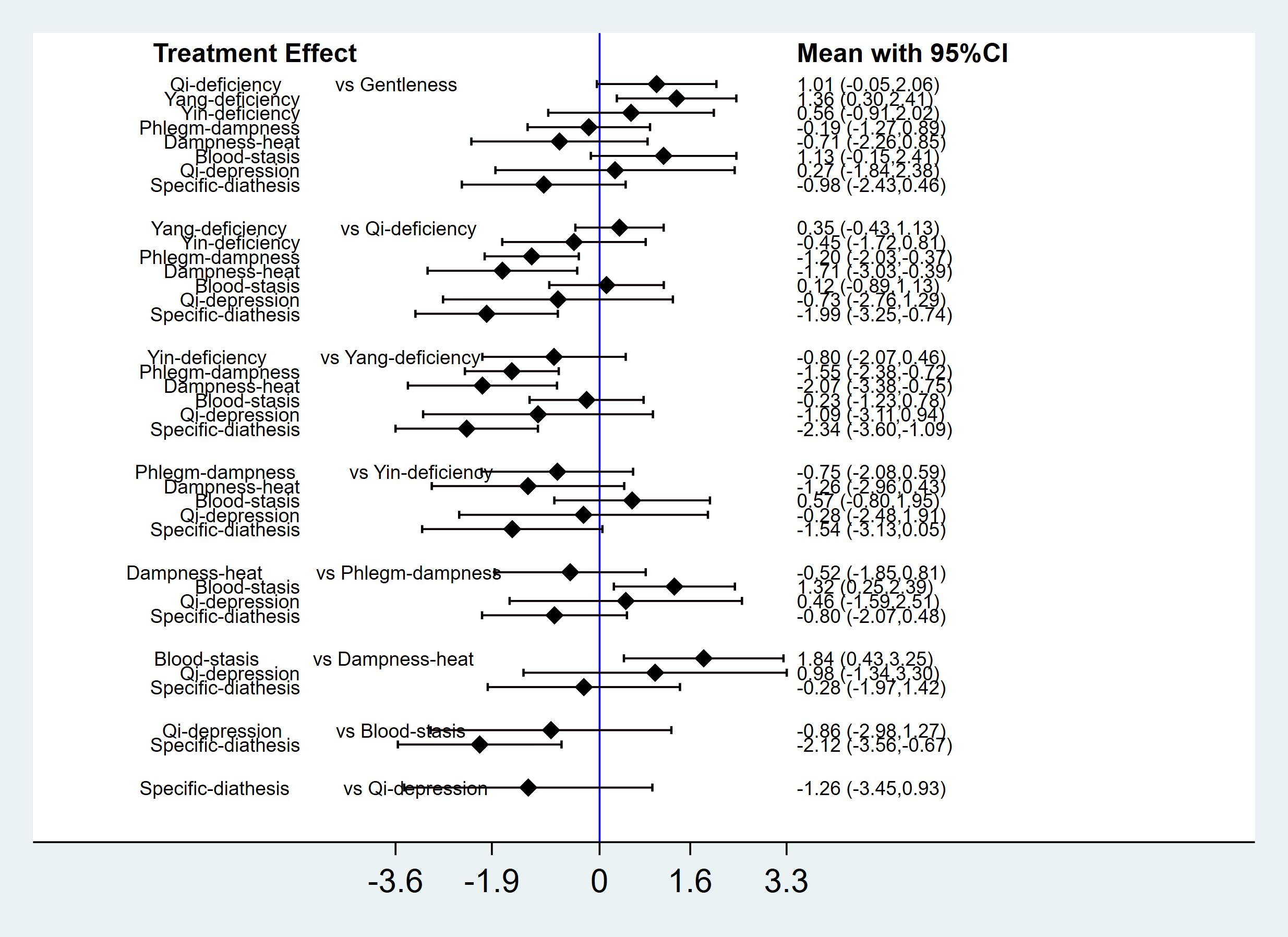


Appendix Fig. 8 Comparison of AE/y between TCM constitutions.

**3.5 CAT score**

A total of 5 studies with 649 participants were included, and 7 kinds of TCM constitutions with 587 participants were analyzed ^[10-13,16-18]^. As shown in Apendix Fig. 7, compared to gentleness constitution, only the CAT score of yang-deficiency was higher with statistical significance (p<0.05). Between the imbalanced constitutions, the CAT score of patients with yang-deficiency was higher than yin-deficiency, phlegm-dampness and dampness-heat, yin-deficiency was lower than qi-deficiency and blood-stasis. There was no statistical difference between other constitutions (p≥0.05).


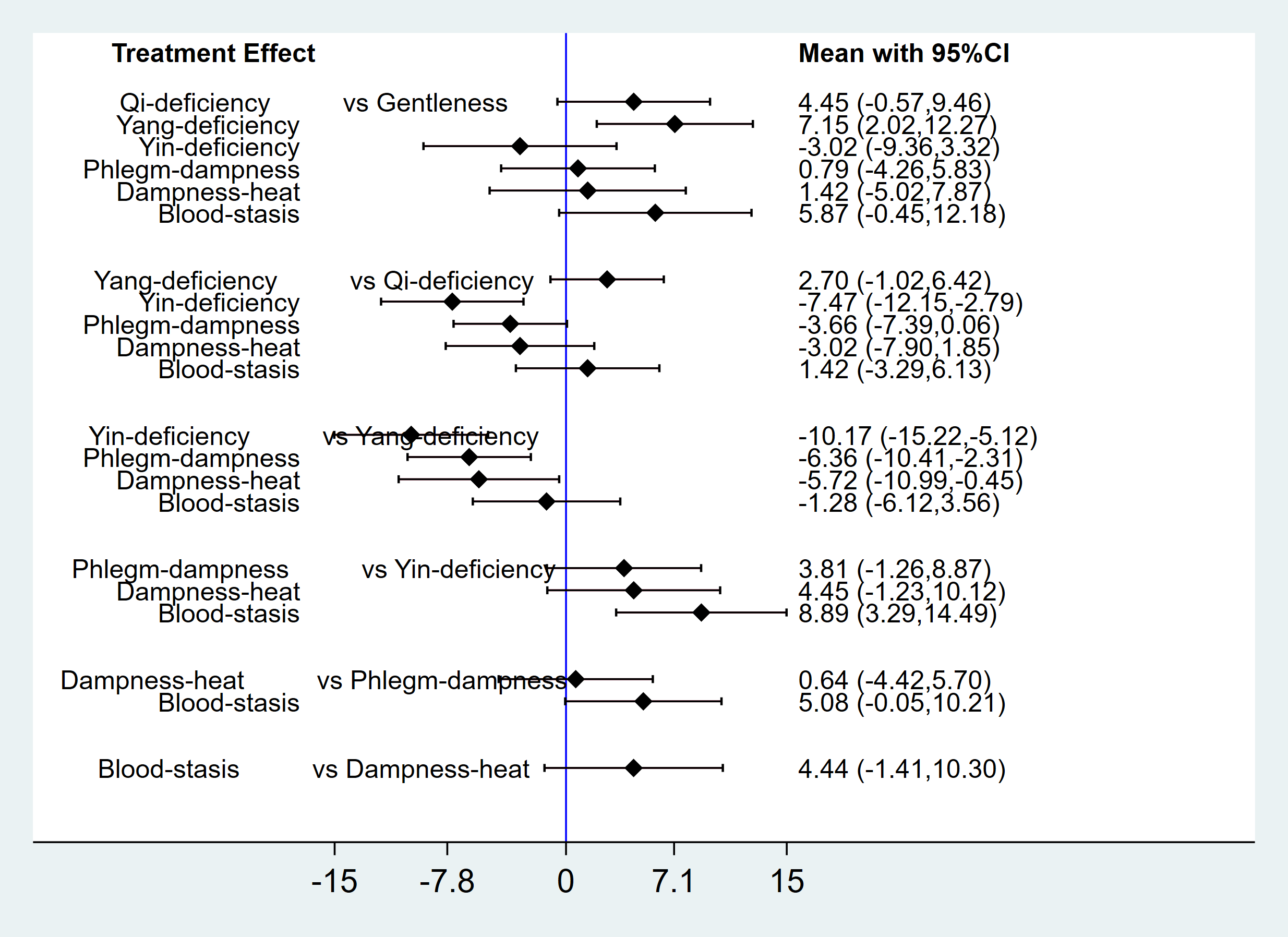


Appendix Fig. 9 Comparison of CAT score between TCM constitutions.

**4. SUCRA of Index**

**4.1 SUCRA of smoking index**

Appendix Fig. 10 SUCRA of smoking index.


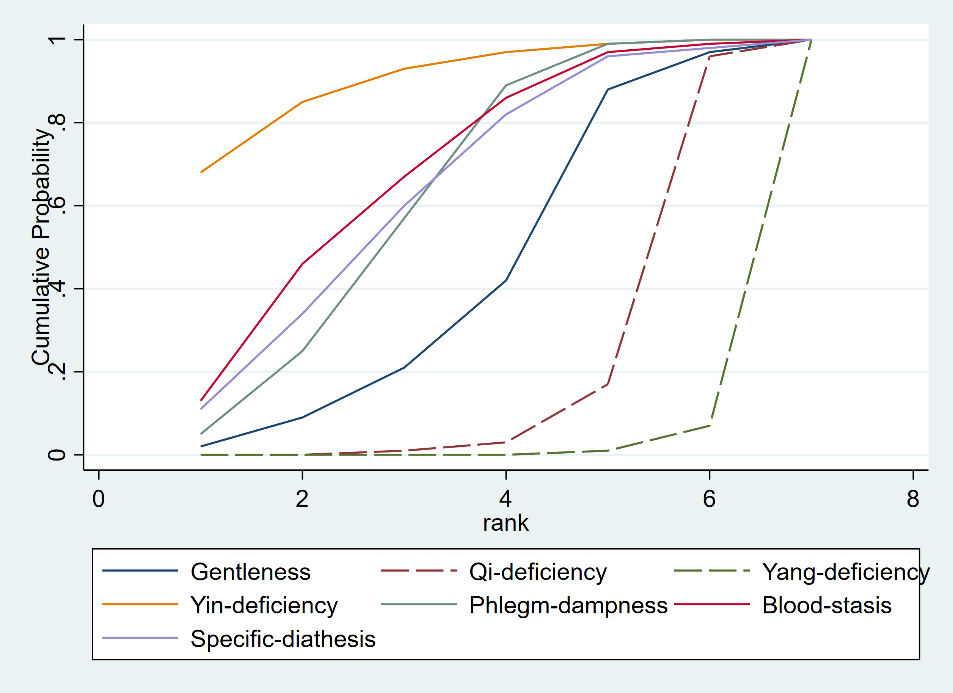
**4.2 SUCRA of** **FEV1/FVC**

Appendix Fig. 11 SUCRA of FEV1/FVC.

**
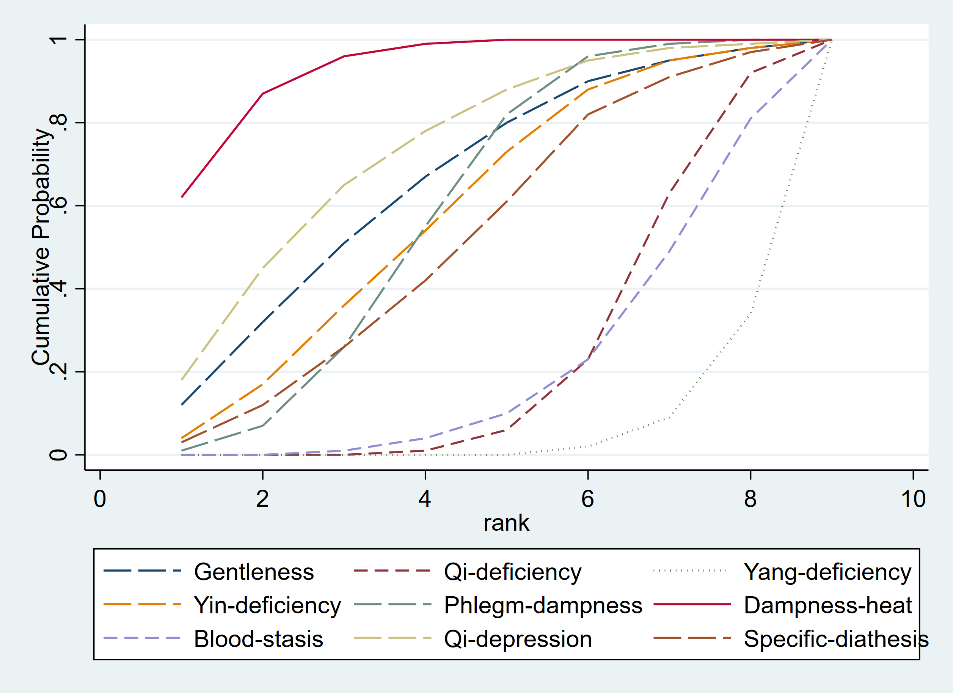
4.3 SUCRA of** **FEV1%Pred**

Appendix Fig. 12 SUCRA of FEV1%Pred.


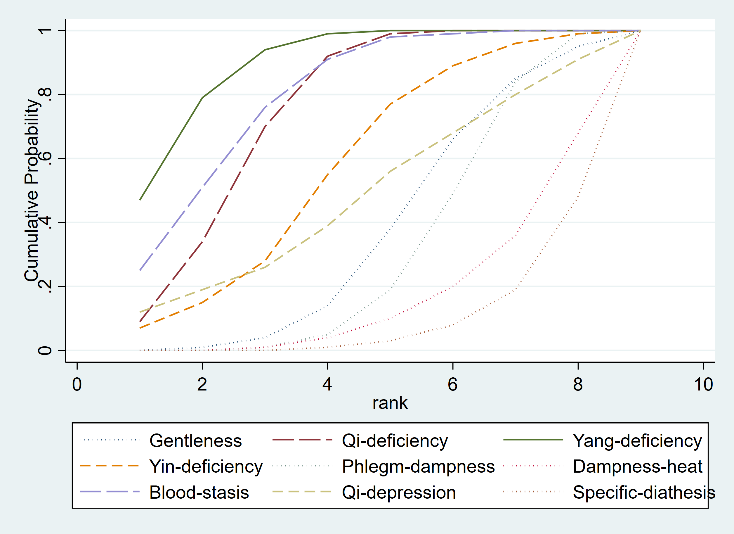
**4.4 SUCRA of** **AE/y**

Appendix Fig. 13 SUCRA of AE/y.

**
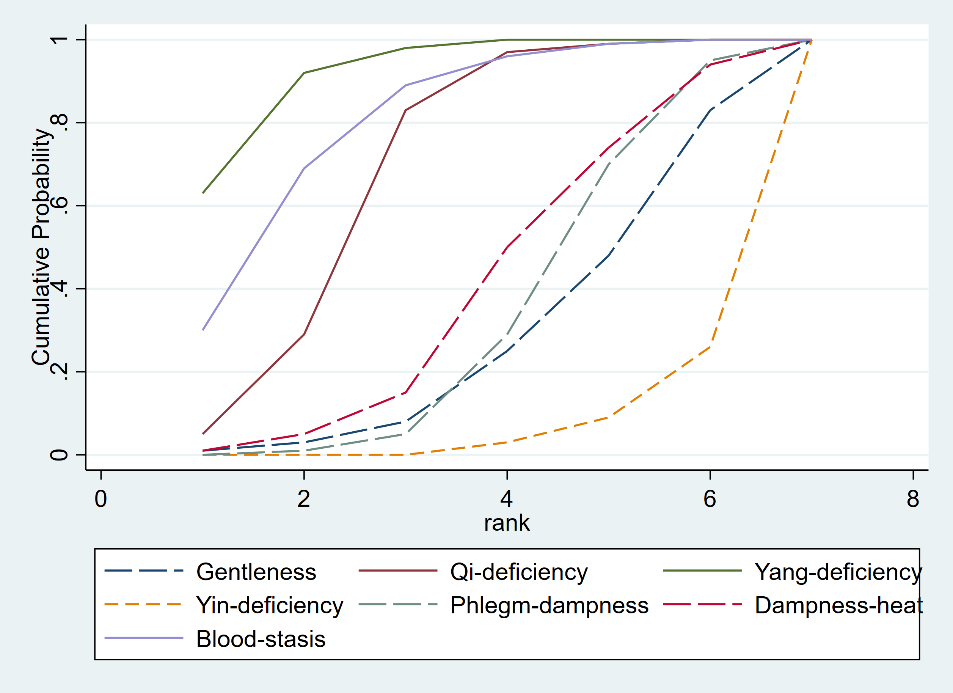
4.5 SUCRA of CAT score**

Appendix Fig. 14 SUCRA of CAT score.

**5. Sensitivity analyses**

**5.1 Smoking index**

Appendix Fig. 15 League table of smoking index.


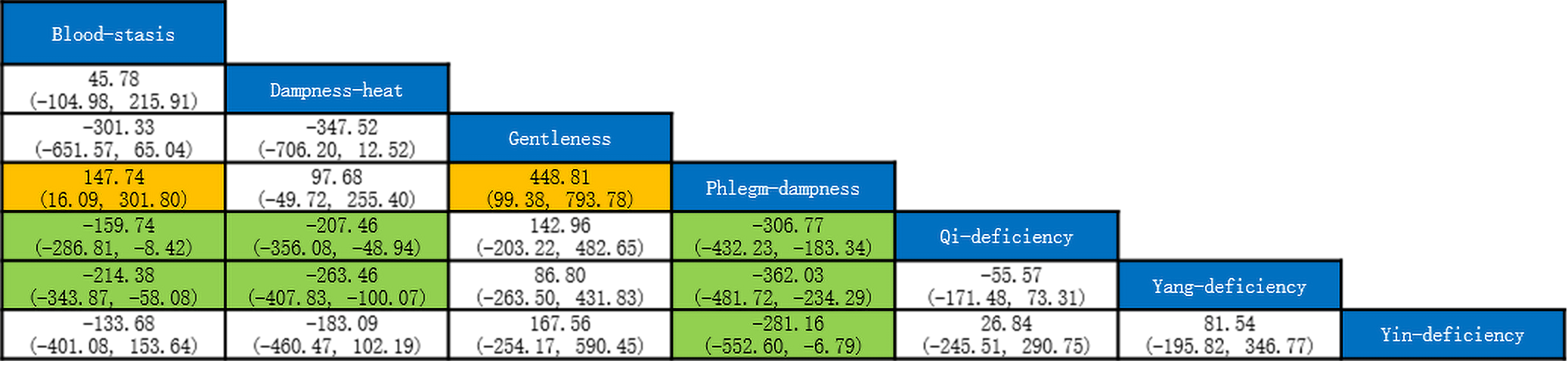


5.2 **FEV1/FVC**


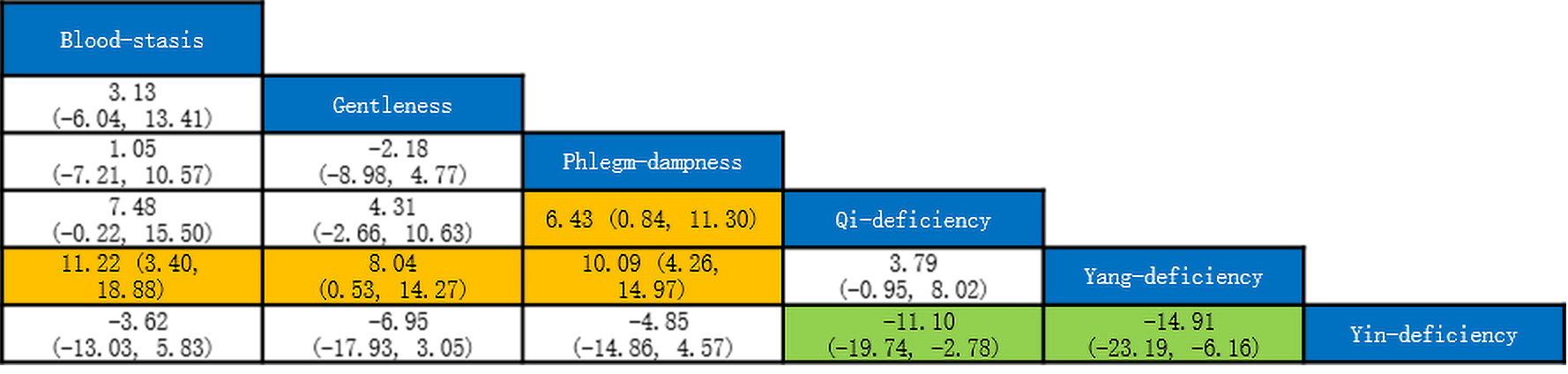


Appendix Fig. 16 League table of FEV1/FVC.


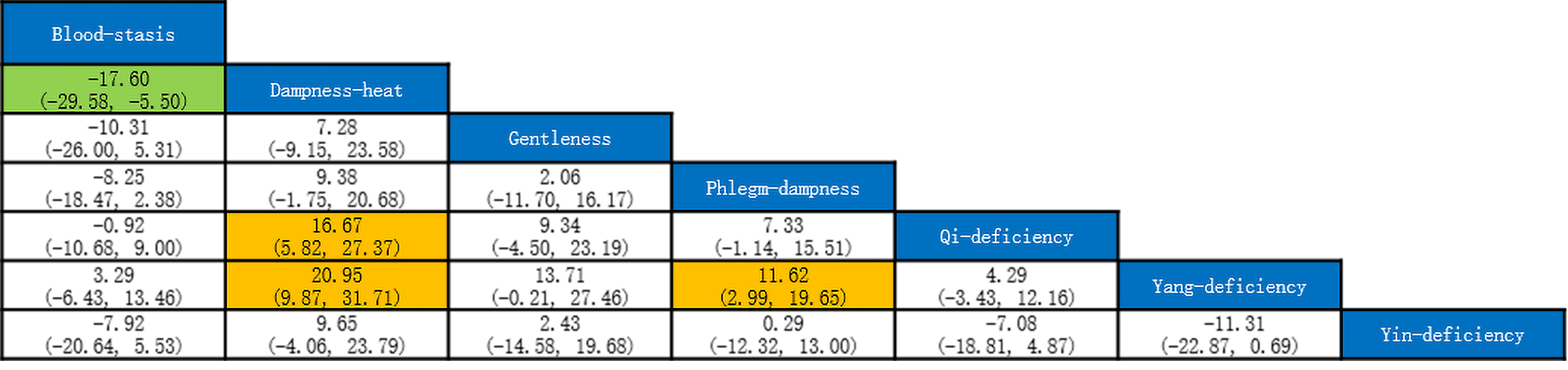


5.3 **FEV1%Pred**

Appendix Fig. 17 League table of FEV1%Pred.


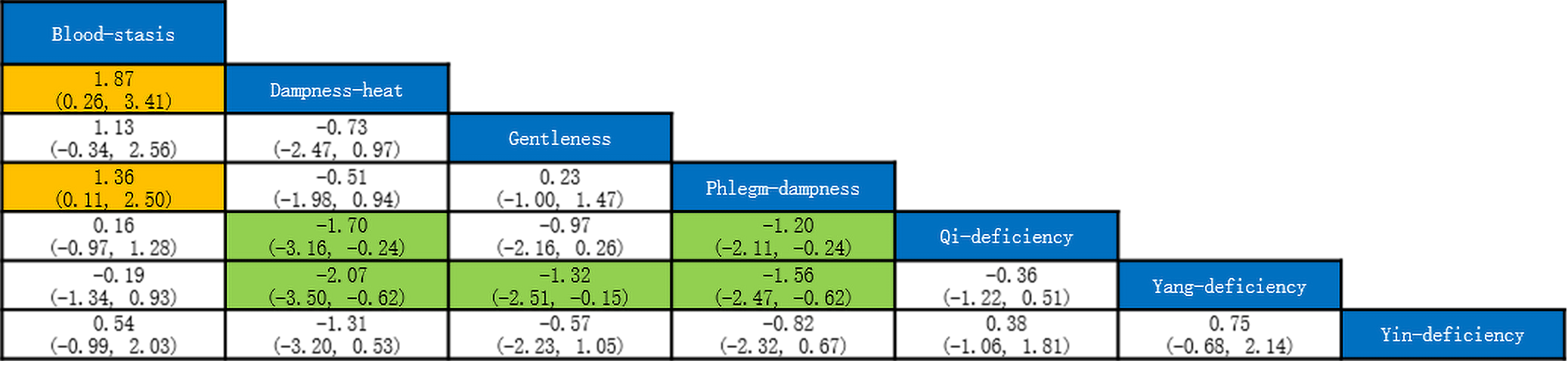


5.4 **AE/y**

Appendix Fig. 18 League table of AE/y.

**6. The framework and direction of interventions**

| TCM constitution | GOLD group* | Possible interventions |
| --- | --- | --- |
| Gentleness | A | Initial single bronchodilator, smoking cessation benefits, rehabilitation trainings target on ventilation-improve |
| Qi-deficiency | E | Initial LABA+LAMA or triple therapy, smoking cessation and vaccination benefit, professional rehabilitation trainings target on ventilation-improve and symptom-control |
| Yang-deficiency | E | Initial LABA+LAMA or triple therapy, smoking cessation and vaccination benefit, professional rehabilitation trainings target on ventilation-improve and symptom-control |
| Yin-deficiency | E | Initial LABA+LAMA or triple therapy, smoking cessation and vaccination benefit, professional rehabilitations training target on ventilation-improve |
| Phlegm-dampness | B | Initial LABA+LAMA, rehabilitation trainings target on ventilation-improve and symptom-control |
| Dampness-heat | B | Initial LABA+LAMA, rehabilitation trainings target on progress-free |
| Blood-stasis | E | Initial LABA+LAMA or triple therapy, vaccination benefits, professional rehabilitation trainings target on ventilation-improve and symptom-control |
| Qi-depression | A or B | Initial single bronchodilator or LABA+LAMA, rehabilitation trainings target on ventilation-improve and emotion-regulation |
| Specific-diathesis | A or B | Initial single bronchodilator or LABA+LAMA, individualized rehabilitation trainings target on ventilation-improve |

Appendix Table. 2 The framework and direction of the interventions based on the conclusions of this study. * Not certain, but a prone possibility.
